# Quantitative and Qualitative Evaluation of Photoreceptor Synapses in Developing, Degenerating and Regenerating Retinas

**DOI:** 10.3389/fncel.2019.00016

**Published:** 2019-02-11

**Authors:** Ryutaro Akiba, Take Matsuyama, Hung-Ya Tu, Tomoyo Hashiguchi, Junki Sho, Shuichi Yamamoto, Masayo Takahashi, Michiko Mandai

**Affiliations:** ^1^Laboratory for Retinal Regeneration, RIKEN Center for Biosystems Dynamics Research, Kobe, Japan; ^2^Department of Ophthalmology and Visual Science, Chiba University Graduate School of Medicine, Chiba, Japan

**Keywords:** photoreceptor synapse, stem cell therapy, circuit reconstruction, retinal degeneration, synapse quantification, ribbon synapse, synaptogenesis

## Abstract

Quantitative and qualitative evaluation of synapses is crucial to understand neural connectivity. This is particularly relevant now, in view of the recent advances in regenerative biology and medicine. There is an urgent need to evaluate synapses to access the extent and functionality of reconstructed neural network. Most of the currently used synapse evaluation methods provide only all-or-none assessments. However, very often synapses appear in a wide spectrum of transient states such as during synaptogenesis or neural degeneration. Robust evaluation of synapse quantity and quality is therefore highly sought after. In this paper we introduce QUANTOS, a new method that can evaluate the number, likelihood, and maturity of photoreceptor ribbon synapses based on graphical properties of immunohistochemistry images. QUANTOS is composed of ImageJ Fiji macros, and R scripts which are both open-source and free software. We used QUANTOS to evaluate synaptogenesis in developing and degenerating retinas, as well as *de novo* synaptogenesis of mouse iPSC-retinas after transplantation to a retinal degeneration mouse model. Our analysis shows that while mouse iPSC-retinas are largely incapable of forming synapses *in vitro*, they can form extensive synapses following transplantation. The *de novo* synapses detected after transplantation seem to be in an intermediate state between mature and immature compared to wildtype retina. Furthermore, using QUANTOS we tested whether environmental light can affect photoreceptor synaptogenesis. We found that the onset of synaptogenesis was earlier under cyclic light (LD) condition when compared to constant dark (DD), resulting in more synapses at earlier developmental stages. The effect of light was also supported by micro electroretinography showing larger responses under LD condition. The number of synapses was also increased after transplantation of mouse iPSC-retinas to *rd1* mice under LD condition. Our new probabilistic assessment of synapses may prove to be a valuable tool to gain critical insights into neural-network reconstruction and help develop treatments for neurodegenerative disorders.

## Introduction

Recent advances in stem cell biology have overturned the long-held belief that neurons do not regenerate. It has now been established beyond doubt that neural networks can be reconstructed after injury or degeneration either by endogenous regeneration (Jorstad et al., 2017; Yao et al., 2018) or by cell or tissue transplantation (Singh et al., 2013; Barnea-Cramer et al., 2016; Mandai et al., 2017). A critical step for neural reconstruction is the requirement for these newly formed or reintroduced neurons to form new chemical synapses. Current methods are however, insufficient to evaluate the extent of neural integration, and more sophisticated methods to evaluate neural integration are in demand.

A chemical synapse is a subcellular structure specialized for communication between neurons through neurotransmitter molecules. As a key parameter to evaluate the functional state of neural networks, various methods have been developed to quantify and assess synapses over the years. The gold standard to assess the state of a synapse is by electron microscopy (EM), where subcellular pre- and post-synaptic components can be directly observed (Geinisman et al., 1996). While EM can provide valuable qualitative information about the state of a particular synapse, it is currently unpractical to survey a large number of synapses, especially if their rough locations are not known. On the other hand, visualization of pre- and post-synaptic markers by immunohistochemistry (IHC) allows for a robust and high throughput analysis, while simultaneously obtaining some qualitative information.

One of the most common approaches to quantifying synapses by IHC is to manually count pre- and post-synaptic marker pairs (Silver and Stryker, 2000; Ribic et al., 2014). Although laborious, a trained expert may be able to reliably count synapses, but different observers may naturally focus on different features and have different thresholds of acceptance. The use of automated software for quantification is another alternative, for example by counting the number of co-localized pre- and post-synaptic markers (Dominic and Eroglu, 2010). These automatic counting programs apparently seem free of human bias, but certain choices are inevitably made by the software developers with or without the user's knowledge. For example, colocalization-based classifiers require binary images where pixels are assigned as stained or unstained for a marker. Binary images are constructed by thresholding the original images by manually adjusting a threshold level (Glynn and McAllister, 2006) or by selecting one of many thresholding algorithms, which calculates a threshold level. In either case, only slight differences in the threshold level can result in drastically different output counts. Different conditions in recording and staining also cause diverse estimates when the same threshold level is applied. A third and more modern approach is machine learning-guided automatic classification methods, which enabled more reproducible analysis (Fantuzzo et al., 2017). However, it is usually unclear what the machine is “learning,” and the factors involved in the decision making of the algorithm are typically unknown.

Above all and most unfortunately, all these synapse quantification methods typically assign a binary value to the marker pairs, either as synapses or not, without accounting for any immature or intermediate properties which however do exist, as exemplified in retinal synaptogenesis, where photoreceptors and bipolar cells form synapses through retinal development (Regus-Leidig et al., 2009). A trained expert can discern these immature or transient states from the morphological, geometrical, and signal intensity properties of an IHC image, allowing for a more nuanced interpretation than the mere number of synapses. Furthermore, there is an increasing need in assessing synapse formation in the field of neural regeneration and cell therapies that involves reconstruction of neural networks by neural cells from endogenous regeneration or transplantation, where the quantitative and qualitative synapse evaluation is considered most relevant to *de novo* neural function. We previously showed that transplantation of mouse ES or iPS derived retinas (mESC/miPSC-retinas) could restore light response in the end-stage retinal degeneration mouse models with some evidence of host-graft synaptic connection (Assawachananont et al., 2014; Mandai et al., 2017; Iraha et al., 2018). A quantitative and qualitative evaluation of synapses would therefore provide a strong clue for estimating the functional potency of grafted tissues, and would further help optimize and develop better conditions for this therapeutic approach.

We thus propose a probabilistic evaluation of synapses from IHC images, which would allow us not only to quantify the number of synapses but also to estimate the likelihood of “synapse-ness” based on multi-synaptic factors on a continuous scale. We named this approach QUANTOS (QUalitative and quantitative ANalysis using Bayes Theorem Optimized for Synapse evaluation). The QUANTOS analysis specializes in the distinctive synapse structure called “ribbon synapse” located between photoreceptors and bipolar cells, namely the first and the second order neurons in the retina. RIBEYE is an essential component of synaptic ribbons found in photoreceptor cells and auditory hair cells of the inner ear. Its molecular structure consists of two domains, one of which is identical to Ctbp2 and is homologous to phosphoglycerate dehydrogenases (Schmitz et al., 2000). RIBEYE is the main component of the synaptic ribbon, which exhibits characteristic horseshoe shape at the photoreceptor axon terminal, and acts as a molecular machinery for efficiently storing and releasing glutamate to the synaptic cleft (tom Dieck et al., 2005; Matthews and Fuchs, 2010). Metabotropic glutamate receptor type 6 (mGluR6) is expressed on dendritic tips of ON-bipolar cells to receive the glutamate released from the photoreceptors (Sterling and Matthews, 2005). We used IHC images of presynaptic RIBEYE and postsynaptic mGluR6 to train QUANTOS and thereby analyzed photoreceptor-bipolar ribbon synapses.

In order to showcase QUANTOS, we first studied the impact of light, i.e., photoreceptor activity on the ribbon synapse formation during development. Electrophysiology was tested in parallel to see the physiological relevance of our synapse assessment. We then used QUANTOS to quantify and assess synaptogenesis of miPSC-retinas after transplantation in the *rd1* mice with end stage retinal degeneration. Here again we tested whether light influences regenerative synapse formation.

## Results

### General Design of the QUANTOS

**Figure 1:**
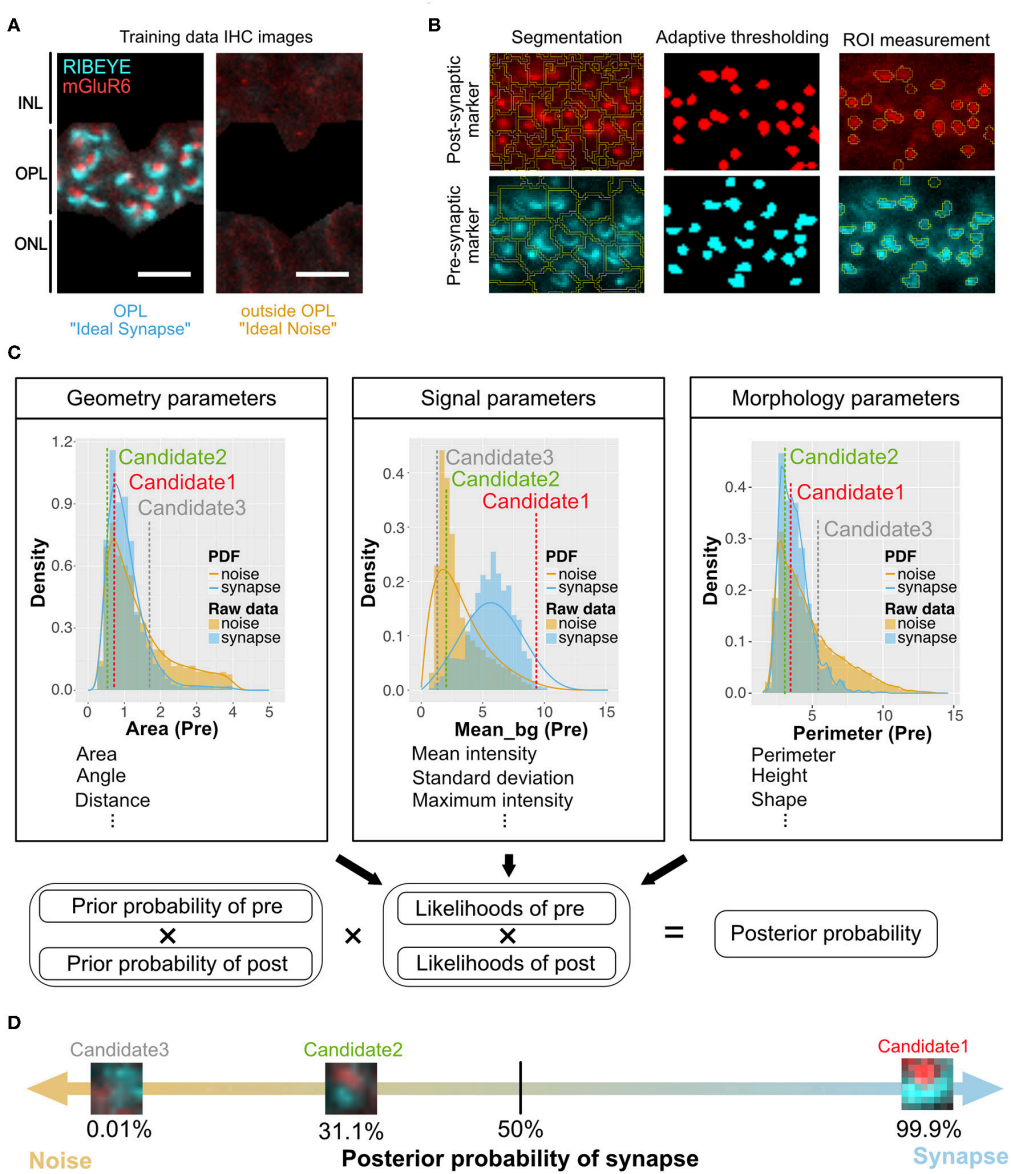
Overview of QUANTOS, a synapse evaluation method using a Naïve Bayes classifier. **(A)** Photoreceptor synapses are visualized by immune-staining of pre-synaptic marker: RIBEYE, and post-synaptic marker: mGluR6. Three to four replicate IHC Images from three P28 B6J mice each were used as training data for *Ideal Synapse* and *Ideal Noise*. The OPL area was manually isolated to train the *Ideal Synapse*, and the area outside the OPL was used to train the *Ideal Noise*. Scale bar = 4 μm. **(B)** IHC images were processed by custom made ImageJ Fiji macros. IHC images were segmented, and thresholded using the background intensity of each segment. The thresholded areas were then overlaid on the original IHC image to extract graphical parameters from ROIs. Details of image processing steps for DAPI, RIBEYE, and mGluR6 are described in Figures S1–S3. **(C)** Upper panel: The distribution of extracted parameters was estimated with either Kernel Density Estimation or Bounded Density Estimation to generate PDFs for *Ideal Synapse* and *Ideal Noise*. These PDFs were used to estimate likelihoods of each synaptic marker. Marker spatial density is used to calculate prior probability. Pre- and post-synaptic markers within 1.2 μm of each other (distance from center of mass) were considered as synapse candidates. Lower panel: Posterior probability of synapse candidates being either synapse or noise is estimated by multiplying prior probabilities and likelihoods of both pre- and post-synaptic markers. **(D)** Posterior probability of being synapses are estimated for each individual synapse candidates. Synapse candidates with more than 50% of posterior probability were classified as synapse. IHC, immunohistochemistry; IPL, inner plexiform layer; OPL, outer plexiform layer; ONL, outer nuclear layer; ROIs, regions of interest; PDFs, probability density functions.

The general design of the method is described in Figure 1 and Figures S1–S5. Samples obtained at different developmental stages of B6J, *rd1*, and *rd1* after miPSC-retina transplantation were co-stained for pre- and post-synaptic markers, Ctbp2 (RIBEYE) and mGluR6 (Figure 1A). Images were segmented and thresholded to isolate regions of interest (ROIs) using macros in ImageJ Fiji (Figure 1B). Image processing protocols were customized for DAPI (Figure S1), RIBEYE (Figure S2), and mGluR6 (Figure S3), respectively, for better detection of ROIs. From each ROI, 34 graphical parameters were extracted using the “Measure” function of ImageJ Fiji (Figure 1B). These parameters can be categorized into 6 *Geometry* parameters, 14 *Signal* parameters, and 14 *Morphology* parameters.

The training data for synapse and noise was generated from IHC images of 3 to 4 replicate slices each from three P28 B6J mice, assuming most of the photoreceptor synapses at this developmental stage would be mature. The outer plexiform layer (OPL), where photoreceptor synapses are formed, was manually cropped to train the *Ideal Synapse* data, and area outside the OPL was used to train the *Ideal Noise* data (Figure 1A). These images were processed as mentioned above, and graphical parameters were extracted to generate probability density functions (PDFs) of *Ideal Synapse* and *Ideal Noise* for each of the parameters. These PDFs were automatically generated by using either Kernel Density Estimation (KDE) or Bounded Density Estimation (BDE). Data were fitted with BDE where there were clear boundaries, and with KDE otherwise. Representative PDFs from each category are presented in the upper panel of Figure 1C (Details of parameters are described in Methods section).

Once PDFs of training data were generated, samples were processed for QUANTOS evaluation. IHC Images were processed to extract pre- and post-synaptic markers ROIs, and their graphical parameters were evaluated against PDFs of *Ideal Synapse* and *Ideal Noise* for estimating likelihoods. Marker spatial density was used for estimating the prior probability of synapse, as higher density of markers results in higher chance of markers being randomly proximal to each other (Figure S4). Pre- and post- synaptic marker pairs within 1.2 μm were considered as “Synapse Candidates”, and their posterior probability was estimated by multiplying the prior probabilities and likelihoods of both pre- and post- synaptic markers altogether (Figure 1C lower panel). This allows QUANTOS to identify the pairs that are more likely to be synapses based on the training data and estimate the total amount of synapses as well as their individual synapse likelihood (Figure 1D). All of these steps were built into ImageJ Fiji Macros and R scripts and uploaded in public repository (https://github.com/matsutakehoyo/QUANTOS).

### Graphical Properties of the Ribbon Synapse

PDFs generated from the training data revealed the properties of noise and synapse staining. The distribution of synapse distances between pre- and post-synaptic markers indicate that synapse distances have a Gaussian distribution with a mean distance of 0.51 μm and a standard deviation of 0.17 μm (Figure S5, upper light panel). From our simulation of random markers, the noise distribution was approximated with a polynomial function of second order; however, noise distances were not necessarily distributed randomly, as noise signals tended to be clustered. The noise angle distribution had a uniform distribution as expected from a random distribution, whereas the synapse angle distribution indicated that the major population of synapses were aligned vertically. Interestingly, the synapse angle distribution had a wide tail indicating synapses at various angles, even horizontally aligned or vertically aligned but in opposite directions (Figure S5, upper light panel). Figure S5 shows the PDFs for *Geometry, Morphology*, and *Signal* features. The synapse *area* distributions had distinctive acute peaks, whereas noise *area* distributions had more larger values for both markers. On the other hand, the *integrated density* was larger in the synapse distribution for both pre- and post-synaptic markers, indicating that noise is either relatively small and bright or large but weakly stained. Many of the *Morphology* parameters, such as *perimeter, width, height, major*, and *minor*, had a broad distribution for noise and a more defined distribution for synapse indicating that noise features are more randomly distributed whereas synapse features do have characteristic staining patterns. Noise distributions for *mean, mode, median, min, max*, and *stdev* parameters tended to have a large peak around small values with a long tail extending to large values. Synapse distributions, on the other hand, were more symmetric and centered around larger values.

### Evaluation of QUANTOS

We evaluated the sensitivity and specificity of QUANTOS, using a data set of synapses on postnatal day (P) 28 and P14, which represent emerging and mature synapses, respectively. These samples were manually evaluated by an expert observer to create a *Ground Truth* to evaluate the performance of QUANTOS.

**Figure 2:**
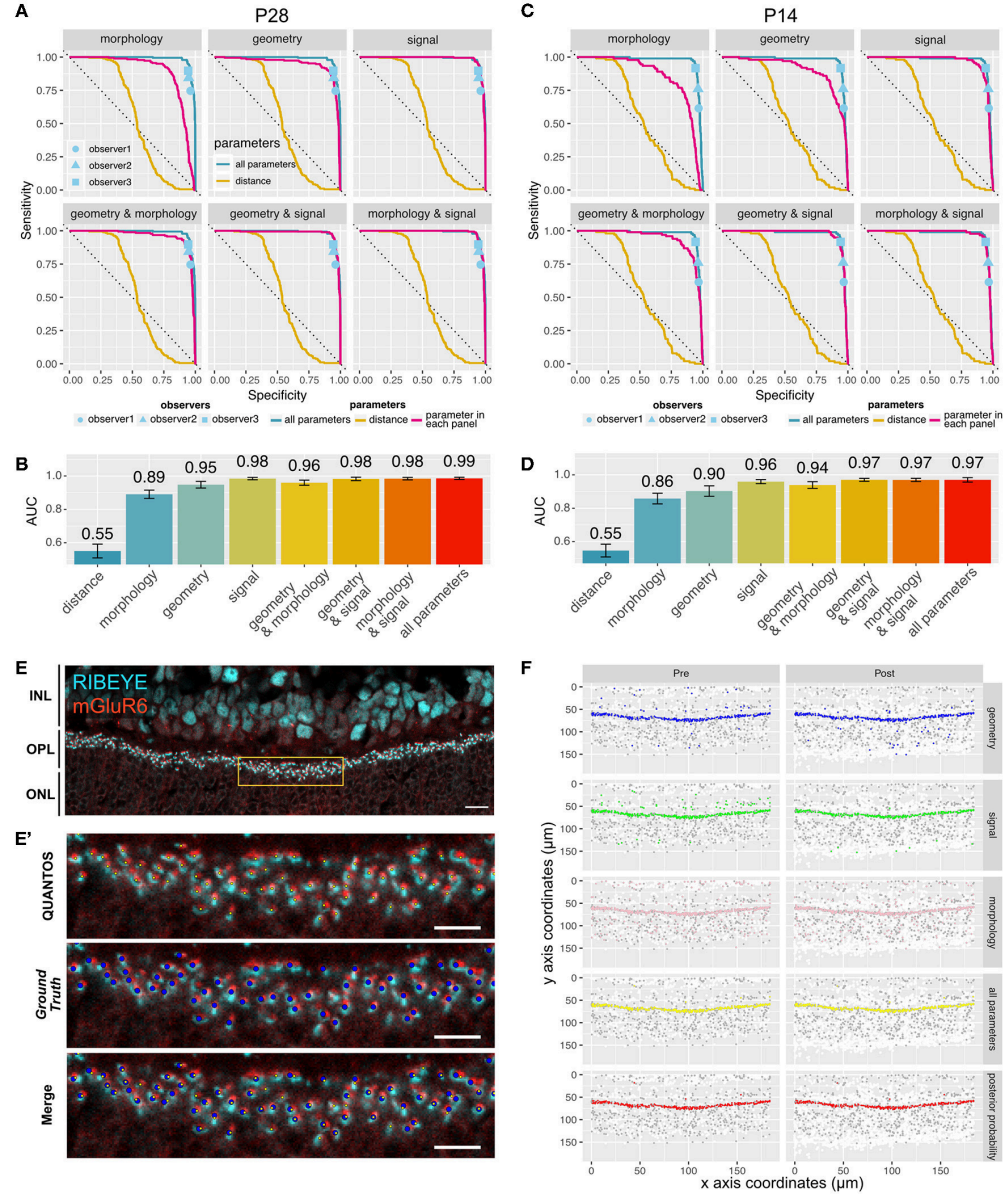
Sensitivity and specificity of QUANTOS. **(A)** ROC curves of classifiers using different combinations of parameters on a P28 sample. ROC curves for each parameter are indicated with a magenta line. The ROC curve of distance and all parameters are shown in all panels for comparison. Dots indicate the results of manual counts by different observers (IHC image: *n* = 1). **(B)** Comparison of AUC between different combination of parameters on P28 sample. Whiskers indicate 95% confidence intervals. **(C)** ROC curves of classifiers using different combinations of parameters on P14 sample (IHC image: *n* = 1). **(D)** Comparison of AUC between different combination of parameters on P14 sample. Whiskers indicate 95% confidence intervals. **(E)** Example of an IHC image of B6J P28 mouse. Yellow box area is shown magnified in **(E')**. Scale bar = 10 μm. **(E')** upper panel: Yellow small dots indicate synapses detected by QUANTOS., middle panel: blue large dots indicate the *Ground Truth* (manually evaluated by an expert), lower panel: overlay image of both QUANTOS results and *Ground Truth*. Scale bar = 5 μm. **(F)** Pre- (left column) and post-synaptic marker (right column) coordinates detected by QUANTOS. Each row shows the synapse candidates, i.e., candidates with high synapse likelihood given different parameters. White dots represent all the markers detected in the Image Processing, and gray dots represents all the synapse candidates (pre- and post-synaptic markers within 1.2 μm), and colored dots represent the synapse candidates with higher likelihood of synapse than noise for different parameters. “all parameters” represents the combined likelihoods of all parameters and pre- and post-synaptic markers. “posterior probability” shows the marker pairs identified as synapses by QUANTOS, which are obtained from “all parameters” by taking into account the prior probability of synapse. ROC, receiver operation characteristics; AUC, area under the curve.

Several receiver operating characteristics (ROC) curves using combinations of *Signal, Morphology*, and *Geometry* parameter categories were generated to better understand the features contributing information to the classifier (Figure 2A). Ground truths for P28 and P14 samples were generated by careful evaluation by an expert observer. For the P28 sample, the ROC curve for the distance parameter alone performed very poorly with an area under that curve (AUC) of 0.55 (95% confidence interval (CI): 0.51–0.58), indicating that the distance-based classifier performance is close to random chance (Figure 2B). Adding *Morphology* and *Geometry* parameters increased the AUC to 0.89 (CI: 0.87–0.92) and 0.95 (CI: 0.93–0.97), respectively. While both parameter categories increased classifier performance substantially, inclusion of *Morphology* seemed to favor sensitivity, whereas *Geometry* enhanced specificity. Among the three categories of parameters, *Signal* parameters showed the largest AUC value of 0.98 (CI: 0.98–0.99), suggesting that *Signal* parameters contained the most information. The best AUC (0.99, CI: 0.98–0.99) was obtained when all the parameters were used (Figure 2C). A similar trend was observed in the P14 sample (Figures 2C,D), where the largest AUC value was 0.97 (CI:0.96–0.98) for the classifier utilizing all the parameters, indicating that QUANTOS was able to reliably evaluate immature synapses in developing retinas.

We also compared QUANTOS against manual counting. Three observers manually counted synapses, and their results were matched with the ground truth for estimating the specificity and sensitivity. QUANTOS outperformed manual counts with a small margin, consistently in both P28 (Figure 2A) and P14 (Figure 2C) images. Manual counting varied in the specificity and sensitivity properties, and the difference was more pronounced on P14, suggesting that human assessment is less stable when encountering immature developmental synapse data. On the other hand, QUANTOS showed robust performance both for immature and mature developmental stages.

The IHC image of P28 C57BL/6J (B6J) (Figure 2E) with the *Ground Truth* (Figure 2E' middle panel) and synapses detected by QUANTOS (Figure 2E' upper panel) are shown for comparison. Also, synapse candidates as evaluated by each parameter category are visualized (Figure 2F). As visualized by the large number of white dots in Figure 2F, the approach of QUANTOS is to pick up as many signals as possible regardless of their intensity, and subsequently filter out candidates based on their likelihoods. This allow us to detect very dim signals and evaluate them accordingly, rather than setting an arbitrary threshold level for markers. Notice that although individual parameter groups may identify markers outside the OPL as synapse candidates, the final synapse evaluation is mostly constrained to the OPL, showing the power of the Naïve Bayes classifier to exclude noise signals by evaluating multiple parameters.

### Quantification of the Photoreceptor Ribbon Synapse During Postnatal Development of B6J Mice Under Different Light Conditions

**Figure 3:**
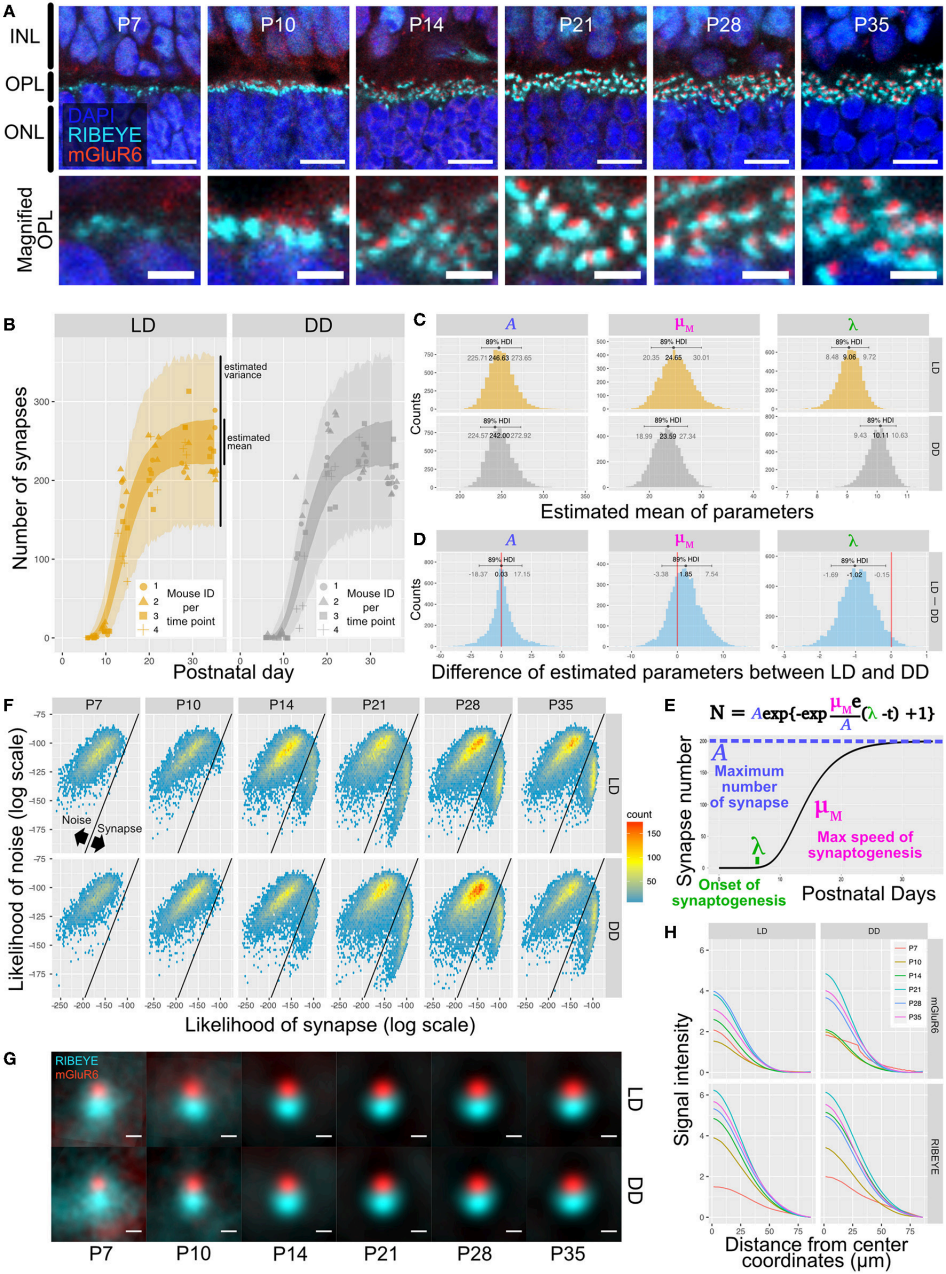
Qualitative and quantitative evaluation of developmental synaptogenesis by QUANTOS in mice reared in DD and LD conditions. **(A)** IHC images of B6J mice on different postnatal days. RIBEYE is the pre-synaptic marker expressed in photoreceptors and mGluR6 is the post-synaptic maker expressed in bipolar cells. Images in upper row show the overview morphology of OPL, and lower row show the magnification of OPL. Scale bar = 10 μm for upper row, 2.5 μm for lower row. **(B)** Result of synapse quantification of postnatal B6J reared under LD and DD conditions. Dots indicate the number of synapses detected in each IHC image. Shape of dots represents the mouse ID for each time point. Dots of each time point were horizontally jittered for better visualization. The dark color-filled area shows the estimated range of mean number of synapses, and the pale color-filled area represents the estimated range of synapse numbers from each IHC image. (*n* = 3 for P7, P10, P35, and *n* = 4 for P14, 21, 28 samples. 3–4 replicates were taken from each mouse as indicated by the shape of markers). **(C)** Posterior distributions of modified Gompertz model parameters with 89% confidence interval. **(D)** Difference of posterior distributions of parameters between LD and DD conditions. **(E)** Developmental synaptogenesis was parameterized with the modified Gompertz's growth curve which has three parameters; the maximum number of synapses **(A)**, maximum rate of synaptogenesis (μ_M_), and the onset of synaptogenesis (λ). **(F)** 2D histograms of all synapse candidates on different postnatal days, with log synapse likelihood on the x axis, and log noise likelihood on the y axis. Synapse candidates on the left-upper side are more likely to be noise, and the ones on the right-lower side are more likely to be synapses. **(G)** All synapses detected by QUANTOS were averaged to visualize the characteristics of synapses on different postnatal days and different rearing conditions. **(H)** Radial profile plots of averaged synapses. The plots show the signal intensity in relation to the center coordinates of pre- and post-synaptic markers. Colors indicate different postnatal days. IHC, immunohistochemistry; INL, inner nuclear layer; OPL, outer plexiform layer, ONL; outer nuclear layer; LD, cyclic light; DD, constant dark; P, postnatal day; HDI, high density interval.

We first used QUANTOS to quantify synapse formation in wildtype B6J mice reared under cyclic light (LD) and constant dark (DD). IHC images of B6J mice on different postnatal days showed that immunoreactivities of RIBEYE and mGluR6 were weak and diffuse on P7 but became stronger on P10. The characteristic horseshoe shape of RIBEYE could be observed after P14, and mGluR6 expression pattern also became punctate on P14. By P21, the shape of the synapse was defined, and the same expression pattern was maintained through P28 and P35 (Figure 3A). QUANTOS detected almost no or very few synapses on P7 and P10 either under LD or DD conditions. From P14 to P21, the number of synapses rapidly increased and remained largely constant through P28 and P35 (Figure 3B). Notably, samples acquired from mice reared in LD condition tended to have more synapses on P10 and P14. We thus modeled the process of synaptogenesis with a growth curve to analyze the effect of light (Figure 3E) using Bayesian parameter estimation. The model shows that while the maximum rate of synaptogenesis (μ_M_) and the maximum number of synapses (*A*) were not significantly different between LD and DD conditions, the onset of synaptogenesis (λ), on the other hand, was faster in LD condition by about one day (Figures 3C,D), indicating that light influences synaptogenesis.

### Quality Changes of the Photoreceptor Ribbon Synapse During Postnatal Development of B6J Mice Under Different Light Conditions

We then inspected the distributions of the likelihoods of synapses determined by QUANTOS in all synapse candidates (Figure 3F). The horizontal axis shows the log synapse likelihood and the vertical axis represents log noise likelihood. The diagonal line represents the boundary where the probability of synapse and noise are equal. On P7, synapse candidates clearly had a peak toward the noise, but from P14 onwards, a second peak with high synapse probability appeared. The synapse peak kept increasing after P21, becoming more prominent on P28 (Figure 3F). Similar trends were observed under both LD and DD conditions.

Lastly, we visualized different states of developmental ribbon synapses by creating average images from all the detected synapses (Figure 3G). RIBEYE and mGluR6 showed diffuse expression patterns on P7 and P10, which became more focused on P14 and later postnatal stages under both LD and DD conditions. The intensity plot against distance from center coordinates of pre- and post-synaptic markers showed that signal intensity became higher at later postnatal stages under both conditions (Figure 3H).

### Effect of Light Assessed by Micro Electroretinography (mERG)

**Figure 4:**
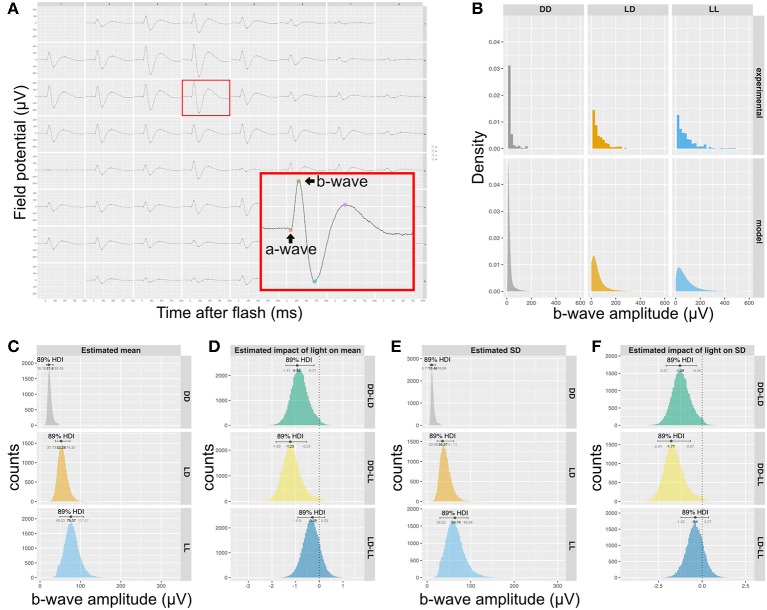
Rearing light conditions alter developmental synaptic function. **(A)** An example of mERG recording. Retinas flat-mounted on the 60-channel probe were stimulated with a mesopic light pulse. The red box is a magnified view of a single channel recording trace, showing a typical waveform with an a-wave and a b-wave. **(B)** Upper panels show histograms of b-wave amplitudes of wildtype P14 mice reared under different light conditions (*n* = 5 for DD and *n* = 4 for LD and LL) and the posterior predictive check of the statistical model used to analyze the data is shown in the lower panels. **(C)** Posterior distributions of mean b-wave amplitude. **(D)** Estimated impact of light on mean b-wave amplitudes. **(E)** Posterior distributions b-wave amplitude SD. **(F)** Estimated impact of light on SD of mean b-wave amplitudes. mERG, micro electroretinography; HDI, high density interval; SD, standard deviation.

In order to determine if the difference in synapse numbers suggested by QUANTOS was physiologically relevant, we recorded the mERG response of P14 retinas, as the difference in synapse numbers was most prominent between LD and DD at this stage. The b-wave, an upward peak between 150 ms after the onset of light pulse stimulation is derived from the ON-bipolar cells that receive signal inputs from photoreceptors through ribbon synapses (Figure 4A). We compared the amplitude of b-waves across mice reared in DD, LD, or constant light (LL) conditions. The histograms of b-wave amplitudes showed a skewed distribution toward smaller values (Figure 4B, upper), with LD and LL having longer tails toward larger b-wave amplitudes. We modeled the data with a hierarchical generalized linear Gamma model (Figure 4B, lower), showing a reasonable summary of b-wave amplitudes. Our model indicates that both the mean value of the b-wave amplitude (Figure 4C), and its standard deviation (Figure 4E) were significantly higher in LD and LL when compared to DD condition (Figures 4D,F). This is consistent with the quantitative evaluation of photoreceptor ribbon synapses in P14 retinas by QUANTOS, suggesting that the presence of light may accelerate development of photoreceptor ribbon synapses.

### Evaluation of Photoreceptor Ribbon Synapses in the Progressive Retinal Degeneration Model (*rd1*) During Development and Degeneration

**Figure 5:**
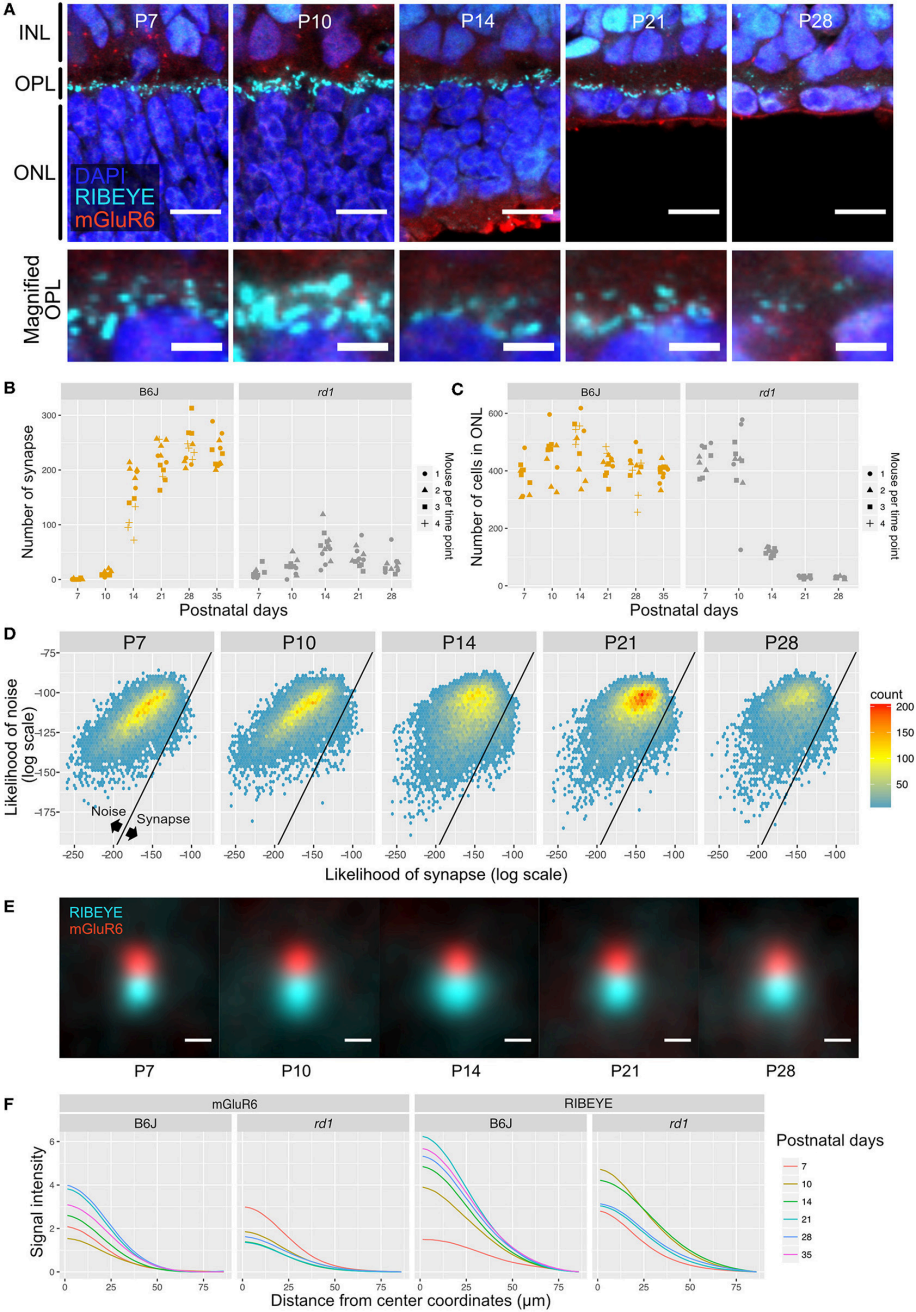
QUANTOS evaluation of synapses during photoreceptor degeneration. **(A)** IHC images of *rd1* mice retinas on different postnatal days. Images in upper row show the overview morphology of OPL, and lower row show the magnification of OPL. Scale bar = 10 μm for upper row, 2.5 μm for lower row. **(B)** The number of synapses detected on different postnatal days in *rd1* mice, accompanied by B6J LD data for comparison (*n* = 3 for each postnatal day of *rd1*. 3–4 replicates were taken from each mouse as indicated by the shape of markers). **(C)** Number of photoreceptor cells estimated form IHC images on different postnatal days of *rd1* mice retinas, accompanied by B6J data for comparison. **(D)** 2D histograms of all synapse candidates on different postnatal days, with log synapse likelihood on the x axis, and log noise likelihood on the y axis. **(E)** Averaged images of all synapses detected by QUANTOS show the characteristics of synapses on different postnatal days and different rearing conditions. Scale bar = 0.5 μm. **(F)** Radial profile plot of averaged synapses. This plot shows the intensity of signals in relation to the center coordinates of pre- and post-synaptic markers. Colors indicate different postnatal days. IHC, immunohistochemistry; INL, inner nuclear layer; OPL, outer plexiform layer, ONL; outer nuclear layer; SD, standard deviation.

One potential application of QUANTOS is to evaluate the synapse formation after transplantation of ES/iPS derived retinas in retinal degeneration models. We first quantified photoreceptor ribbon synapses in *rd1* mouse retinas, in which rod photoreceptors are mostly lost in the first 4 postnatal weeks. IHC images of *rd1* retinas on P7 showed weak expression of RIBEYE, which became prominent on P10, showing some horseshoe shape patterns (Figure 5A). However, the RIBEYE expression decreased from P14, leaving almost no signals by P28. On the other hand, the expression of mGluR6 was constantly weak throughout all postnatal stages. The number of synapses quantified by QUANTOS are shown in Figure 5B together with the B6J data (LD condition) for comparison. Results of *rd1* retinas quantification showed a slight increase of synapses from P7 to P14, followed by a gradual decrease thereafter. The number of synapses in *rd1* was dramatically reduced by P28 compared to B6J. The number of ONL cells started decreasing on P14 and continued to decrease through P21 and P28 (Figure 5C). ONL cells did not completely disappear even on P28, as cone photoreceptors survive longer than rods (Lin et al., 2009).

Next, we generated 2D histograms of synapse and noise log likelihoods for all the synapse candidates. While synapses seemed to increase toward P14 in *rd1*, no distinctive synapse group was observed as in the wildtype, suggesting that synapses formed in *rd1* are incomplete and small in number compared to B6J (Figure 5D). We again visualized synapses from different postnatal days by averaging all the synapses detected by QUANTOS (Figure 5E). The signal of RIBEYE transiently increased in size on P14, but then continuously decreased through P21 and P28. mGluR6 expression did not noticeably change from P7 to P21, but slightly decreased on P28. Signal intensity of averaged synapses were plotted against the distance from the center of synaptic markers (Figure 5F). These plots show that the intensity peak became higher on later postnatal days in B6J, but the opposite trend was found in *rd1* mice, showing lower intensity on later postnatal days for both synaptic markers.

### Quantity and Quality Change of Ribbon Synapses After Subretinal Transplantation of miPSC-Retinas Into *rd1* and the Effect of Light on Regenerative Synaptogenesis

**Figure 6:**
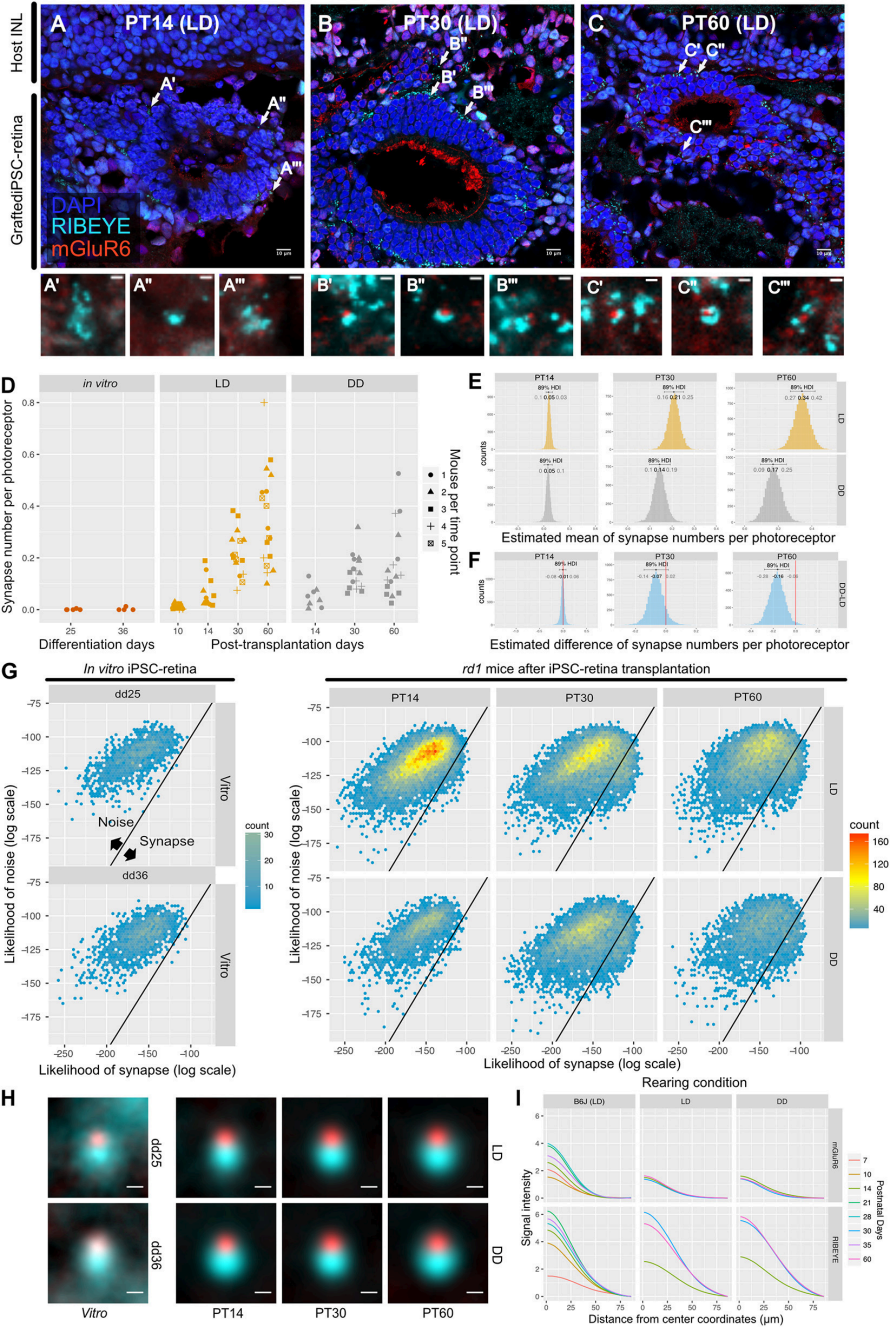
QUANTOS detects *de novo* synapses after miPSC-retina transplantation and shows that light enhances synaptogenesis. **(A–C)** Example IHC images of *rd1* mice after miPSC-retina transplantation on PT 14, 30, 60. Bottom panels show magnified images of some synapse candidates. Scale bar = 10 μm **(D)** Number of synapses of *rd1* mice before and after transplantation of miPSC-retina under different rearing light conditions. (5 and 4 retinal organoids were sampled for *in vitro* dd25 and dd36, respectively. *n* = 4 for PT10 LD, *n* = 3 for PT14 LD, *n* = 2 for PT14 DD, *n* = 5 for PT30 LD, *n* = 4 for PT30 DD, *n* = 5 for PT60 LD, *n* = 4 for PT60 DD. 3–4 replicates were taken from each mouse as indicated by the shape of markers). **(E)** Estimated mean number of synapses per photoreceptor on PT 14, 30, and 60. **(F)** Difference of estimated mean number of synapses per photoreceptor between DD and LD. **(G)** 2D histograms of all synapse candidates on different postnatal days, with log synapse likelihood on the x axis, and log noise likelihood on the y axis. **(H)** Average synapse of *rd1* mice before and after miPSC-retina transplantation. All synapses detected by QUANTOS were averaged from different time points, respectively. Scale bar = 0.5 μm. **(I)** Radial profile plot of averaged synapses. This plot shows the intensity of signals in relation to the center coordinates of pre- and post-synaptic markers. Colors indicate different postnatal days. Data of B6J is presented together for comparison. IHC, immunohistochemistry; PT, post-transplantation day; LD, cyclic light; DD, constant dark; dd, differentiation day; INL, inner nuclear layer; OPL, outer plexiform layer, ONL; outer nuclear layer.

We transplanted miPSC-retinas of differentiation day (dd) 12–13 into 9 to 12-week-old *rd1* mice. We then investigated synaptogenesis by IHC on post-transplantation days (PT) 14, 30, and 60 (approximately equivalent to dd26, 42, and 72). IHC images from PT14 retinas showed immature expression of RIBEYE and almost no expression of mGluR6 (Figure 6A). On PT30 and PT60, typical horseshoe shaped RIBEYE and punctate mGluR6 immunoreactivities were observed surrounding the transplants, suggesting the formation of synapses (Figures 6B,C). We first examined dd25 and dd36 samples by QUANTOS to test if miPSC-retinas could form synapses *in vitro*, and found that there was almost no synapse formation *in vitro* regardless of differentiation day (Figure 6D, left). In contrast, a substantial number of synapses was formed in the post-transplantation *rd1* retinas (Figure 6D, middle). The number of synapses per graft photoreceptor increased substantially from PT14 to PT30 and then to PT60, indicating that transplanted photoreceptors form new synapses as miPSC-retinas integrate and mature in the host *rd1* retinas. We also tested the effect of light on post-transplantation synaptogenesis and found that the number of synapses per photoreceptor was higher in LD condition on PT60, indicating that that similarly to developmental B6J retina, light resulted in an increased number of synapses (Figures 6D–F). 2D histograms of noise and synapse likelihoods of *in vitro* miPSC-retinas showed sparse synapse candidates distributed in the noise region, and almost no candidates toward synapse were observed on dd25 and dd36 samples (Figure 6G). After transplantation, synapse candidates were observed mostly toward noise on P14, with LD-conditioned mice having more candidates toward synapse. On P30, a small peak was observed toward higher synapse likelihood in both LD and DD. The small synapse peak remained in the LD samples on PT60 but was less prominent in the DD condition.

For visualization of the expression pattern, all synapses detected by QUANTOS were averaged (Figure 6H). Before transplantation, the expression pattern of RIBEYE from *in vitro m*iPSC-retinas was diffuse on dd25 but became more focused on dd36. mGluR6 expression was quite weak both on dd25 and on dd36. After transplantation, the expression pattern of RIBEYE became larger and brighter, but mGluR6 expression was relatively weak on all post-transplantation days. This trend was confirmed by intensity plots (Figure 6I). The intensity of RIBEYE became higher on later post-transplantation days, but the intensity of mGluR6 was low throughout all time points, when compared to the B6J LD condition.

### Mature/Immature Likelihoods of Synapses

We built a synapse classifier using synapse and noise training data. Similarly, we attempted to further discriminate synapses by training a new classifier with P10 (DD) and P28 (LD) synapses, representing relatively immature and mature synapses, respectively. Sample images of B6J P14 and P21 synapses are shown (Figures S6A,C), along with the results of QUANTOS evaluation (Figures S6B,D), showing examples of mature and immature synapses.

**Figure 7:**
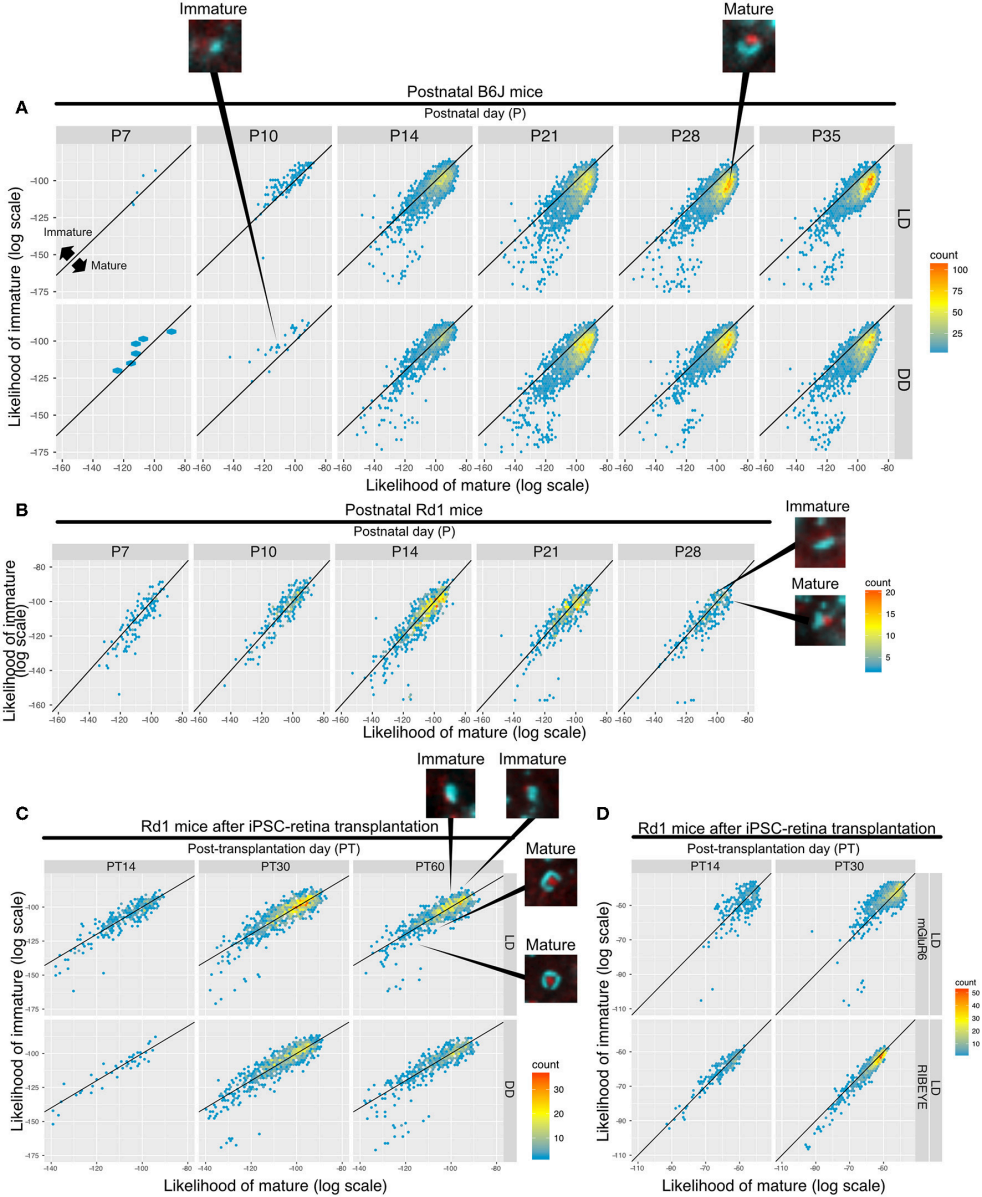
QUANTOS can compare the relative maturation of synapses formed during development, degeneration, and regeneration of the retina. **(A–C)** 2D histograms showing the log likelihood of mature synapse on the x axis, and the log likelihood of immature synapse on the y axis. **(A)** Synapse maturation of B6J mice reared under LD or DD conditions with representative IHC images of synapses from P10 DD and P28 LD. **(B)** Synapse maturation of *rd1* mice with representative IHC images of mature and immature synapses are presented as examples. **(C)** Synapse maturation of *rd1* mice after miPSC-retina transplantation with representative IHC images of mature and immature synapses. **(D)** Synapses of *rd1* mice after miPSC-retina transplantation with pre- and post-synaptic maker log mature/immature likelihoods displayed separately. IHC, immunohistochemistry; LD, cyclic light; DD, constant dark.

Samples of B6J during development under DD/LD conditions and *rd1* after transplantation of miPSC-retina were tested for maturity. 2D histograms of mature and immature synapse likelihoods of the B6J mice show an immature small population dominantly on P10 which starts to shift toward the mature region in the LD condition on P14, but it is delayed in the DD condition (Figure 7A). This suggests that LD synapses acquire mature properties earlier than DD synapses. The majority of the synapses were classified on the mature side by P21 in both LD and DD conditions. Synapses in postnatal *rd1* mice exhibited a mixture of mature and immature properties (Figure 7B). Synapses in *rd1* mice after miPSC-transplantation were more diverse in mature/immature likelihoods, but some showed higher mature likelihood (Figure 7C). When the log likelihoods of mature and immature synapse were plotted separately for pre- and post-synaptic markers, the pre-synaptic marker population is shifted toward mature from PT14 to PT30, suggesting expression of more mature RIBEYE in iPSC-retina after transplantation (Figure 7D).

## Discussion

In the present study, we introduced our new synapse evaluation method using a Naïve Bayes classifier, “QUANTOS,” which offers a transparent evaluation of multiple parameters, thereby achieving a reproducible and robust counting of retinal ribbon synapses. Many synapse classifiers are simply colocalization-based, however spatial information alone is not enough to reliably evaluate synapses. Our data indicate that the mean distance between pre- and post-synaptic markers in the photoreceptor ribbon synapse is 0.51 μm, which is consistent with a previously reported distance of 400 to 800 nm for mGluR6 and presynaptic active zone in an EM study (Vardi et al., 2000). The identification of overlapping markers depends largely on the adjustment of the threshold which is arbitrary and unstable as evidenced by the ROC curve of the distance-based classifier, which showed near random chance performance. In manual counting, the counter is evaluating multiple parameters simultaneously and setting thresholds of acceptance for those features based on the present image and prior experience. These assessment criteria are often difficult to articulate, and different observers may place importance on different features. Our present approach is more transparent as all the parameter PDFs are defined, and the user may trace back the features that contribute to a particular synapse assessment. QUANTOS allows users to see which parameters and how those parameters are changing during synaptogenesis, and what is causing the difference between synapse and noise, or mature and immature synapses.

We originally intended to only use parameters that had large differences in their synapse and noise PDFs. However, ROC analysis revealed that the largest AUC is obtained when all the parameters are employed. In fact, while the *Signal* parameters alone have an excellent AUC, the *Morphology* and *Geometry* parameters combined also have a considerable AUC, indicating that features other than the signal parameters also contain valuable information. Even when the difference between synapse and noise PDFs is small for individual parameters, they may contain significant information when combined. Another advantage of using a large number of parameters is that QUANTOS is more robust as it is less reliant on a particular trait. This robustness is well-illustrated by the selection process of the synapse, where many parameters detect different synapse candidates, but only the ones in the OPL were selected in the end (Figure 2F). In practical comparison, it is interesting that manual counts fall very near along the ROC curve, indicating that QUANTOS is making a very similar trade-off between sensitivity and selectivity to a human observer; however, as its parameters are well-defined, it is highly reproducible, unlike manual counts.

QUANTOS detects synapses based on the *Ideal Synapse* training data which was obtained from the OPL. An important caveat to this is that both rod and cone synapses are contained in the training data. Cones also form ribbon synapses and express RIBEYE and mGluR6, similarly to rod ribbon synapses. Therefore, QUANTOS cannot currently distinguish between rod and cone synapses. Rod photoreceptors account for about 97% of all photoreceptors (Jeon et al., 1998), and therefore the training data of QUANTOS should consist mostly of rod synapses with a small fraction of cone synapses. PDFs of *Ideal Synapse* data did not show any discrete peaks for cone synapses, indicating that either they largely overlap with rod characteristics or the fraction of cone synapses is too low to contribute. We plan to use cone pedicle specific markers to overcome this limitation in future studies.

The noise/synapse classification revealed two separate populations (Figure 3F); the mature/immature classifier, on the other hand, did not reveal such discrete groups (Figure 7). This indicates that immature and mature synapses exist on a continuum with no clear boundaries, at least regarding their IHC properties. While our definition of mature and immature synapses is completely arbitrary, we were still able to observe a shift from more immature to more mature synapses along retinal development, which would have been impossible to observe with traditional synapse classifiers. Furthermore, the characterization of mature/immature synapse properties by QUANTOS was also consistent with the reported developmental features of the photoreceptor ribbon synapse. An EM study reported that development of the photoreceptor ribbon synapse starts around P4, but the photoreceptors only form dyads with horizontal cells at this stage. Later, around P7–P14, dendrites of bipolar cells invaginate into the dyad and make triads, which makes the photoreceptor ribbon synapse complete. The number of triads starts increasing from P7 to P14, and shows a gradual decrease afterwards (Blanks et al., 1974). Another IHC study showed that the number of mGluR6 puncta rapidly increases in the first two postnatal weeks, and plateaus around P21 (Anastassov et al., 2017). Consistent with these reports, we found that the majority of the synapses were classified as mature by P21.

Furthermore, we used QUANTOS to quantify the photoreceptor ribbon synapses formed under different light conditions in relevance to the physiological function of photoreceptors. There are conflicting reports on the effect of light on photoreceptor synapses. In adult mice, photoreceptor ribbon synapses increase with continuous illumination when observed by EM, but this is thought to be illumination-dependent detachment of ribbons from the active zone, and not an increase of synapse numbers (Spiwoks-Becker et al., 2004). Another study using ERG reported that on P30 and P60 a- and b-wave amplitudes decrease in DD reared mice when compared with LD reared mice (Tian, 2004). Another mERG study reported that dark rearing does not affect rod-driven b-wave amplitude (Dunn et al., 2013). Our analysis of the effect of light throughout the synaptogenesis period suggests that light is accelerating synapse formation itself. Our mERG recordings support the QUANTOS synapse analysis. These findings suggest that the number and/or function of photoreceptor synapses is enhanced by light. This could be in part due to the delayed maturation of the retinal cells, since it was previously reported that morphological maturation of bipolar cells is delayed by dark rearing (Wu and Chiao, 2007). This highlights the sensitivity of QUANTOS, as raw images do not appear noticeably different at first glance. Despite the fact that there is quite a lot of variance between mice, we were able to identify the differences using QUANTOS.

Lastly, we assessed synapse degeneration in the retinal degeneration model *rd1* and regenerative synaptic formation/maturation of transplanted miPSC-retinal tissues. Previous EM studies indicate that early development of *rd1* is normal up to about P10, but later bipolar cell dendrites fail to invaginate photoreceptors (Blanks et al., 1974). The number of synapses on P28 was dramatically reduced when compared with B6J mice, and the remaining synapses had low likelihood of synapse and low likelihood of being mature as determined by QUANTOS, suggesting incomplete synapse formation, consistent with past EM studies.

Although miPSC-retina formed no substantial synapses *in vitro*, the number of synapses seemed to increase in a time dependent manner after transplantation, suggesting that these synapses are not the remaining *rd1* host synapses, but newly-introduced synapses formed in transplanted cells. This result indicates that synaptogenesis requires intra-ocular factors that are not present in the *in vitro* environment. This was consistent with a recent study reporting that *in vitro* miPSC-retinas can mature up to an equivalent stage of P6 wildtype retina, but do not show apparent synaptogenesis (DiStefano et al., 2018). Here again, the number of synapses formed after transplantation was enhanced by light, suggesting a positive effect of some visual stimuli after transplantation to boost synapse formation.

Finally, a major limitation of the current implementation of QUANTOS is that it is based on thin sliced section samples, with virtually no information on the z-axis. In addition to compromising z-axis information, section preparation inevitably results in some synapses sliced at various angles resulting in an incomplete representation. We are currently expanding QUANTOS to process stacks of 2D confocal images in 3D to analyze whole-mount images. This would also allow a more complete evaluation of complex tissue, such as transplanted retinas.

## Conclusion

We have established an innovative method, that we have named QUANTOS, to robustly and transparently evaluate the quality and quantity of the photoreceptor ribbon synapse from IHC images. Using this method, we have successfully evaluated developmental synaptogenesis of the wildtype B6J mouse retina, the degenerative process of the *rd1* mouse retina, and regenerative synaptogenesis of the miPSC-retina after transplantation. We showed that miPSC-retina cannot form substantial *de novo* synapses *in vitro* but it is capable of extensive synaptogenesis after transplantation. We also showed that light has a positive effect both on the quantity and quality of synapses formed during developmental and regenerative synaptogenesis of photoreceptors. Although QUANTOS was optimized for the photoreceptor ribbon synapse in this study, this method can be easily adapted to observe synaptogenesis of other neurons.

## Methods

### Animals

All animal experiments were conducted in accordance with local guidelines and the ARVO statement on the use of animals in ophthalmic and vision research. All the experimental protocols were approved by the animal care committee of the RIKEN Center for Biosystems Dynamics Research (BDR).

C57BL/6J (B6J) mice were used for developmental analysis, and C57BL/6J-Pde6b^*rd*1−2*J*^/J (*rd1*) mice were used for the retinal degeneration model. Enucleation was carried out immediately after sacrificing the animals.

Animals were reared under different illumination conditions to investigate the effect of light on synaptogenesis. In the LD condition, animals were kept under the standard 12 h light (from 8 a.m. to 8 p.m.) and 12 h dark cyclic light environment. The light source was a fluorescent light bulb with an irradiance, measured vertically upward from the bottom of the rearing cage, of 67.4 μW/cm^2^ (233l ux). For the DD condition, B6J mice were kept in constant darkness from before birth. For the retinal transplantation experiments, *rd1* mice were maintained in LD condition and then moved to DD condition immediately after transplantation. For the DD condition, all the animal care was carried out using LED lights with peak wavelength of 690 nm, which had a minimal effect on mouse photoreceptors.

Additionally, animals were reared in constant light (LL) condition for micro electroretinography (mERG) analysis. The irradiance was the same as the LD condition, but the light was always kept on in this condition.

### Differentiation and Subretinal Transplantation of iPSC-Retinas

The *Nrl*-GFP miPSC line was generated from transgenic *Nrl*-eGFP mice (Akimoto et al., 2006; Homma et al., 2013). The Ctbp2:tdTomato fusion protein was expressed under Nrl promoter by introducing the gene on the ROSA 26 locus of these lines as previously described and characterized (Mandai et al., 2017). Maintenance, differentiation and optic vesicle structure preparation for transplantation was as previously described (Assawachananont et al., 2014). Briefly, optic vesicle structures (dd 12–13) were cut to small pieces (around 0.5 mm × 2 mm), on the day of transplantation, and inserted sub-retinally into the eye of the 9-12-week-old *rd1* mice using a glass micropipette with a tip diameter of ~ 500 μm. Indomethacin (10 mg/L) was added to the drinking water of all transplanted mice starting on the day of transplantation.

### Immunohistochemistry *(*IHC*)*

Animals were sacrificed by cervical dislocation and the eyes were enucleated. The eyes were perforated using a 22G needle, and fixed with 4% paraformaldehyde (PFA) for an hour and then hemisected followed by cryo-protection with 30% sucrose in phosphate buffered saline (PBS) over night at 4°C. The fixed eyes were embedded in OCT compound (4583, Sakura Finetek Japan, Tokyo) and stored at −30°C. Cryo-sections of 12-μm thickness were made with a Cryostat CM3050S (Leica). Heat induced antigen retrieval was carried out at 100°C for 20 min using citrate buffer (10 mM sodium citrate, pH 6.0). The antigen retrieval process removes fluorescence of all fluorescent proteins. Samples were then blocked with Blocking One (nacalai tesque) with 3% Triton X-100 at room temperature for 1 h. Samples were next incubated with primary antibodies in 3% Triton X-100/Dako REAL Antibody Diluent (S2022, Dako, Denmark) over 3 nights at 4°C, followed by washing with PBS 5 times. For the primary antibody of the pre-synaptic marker, we used mouse anti-CtBP2 (612044, BD biosciences, Franklin Lakes, NJ, USA). For the primary antibody of the post-synaptic marker, rabbit anti-mGluR6 antibody (AGC-026, Alomone labs, Jerusalem, Israel) was used. Altogether, expressions of RIBEYE and mGluR6 in proximal area were highly indicative of a functional photoreceptor-ON bipolar cell ribbon synapse.

Samples were incubated with secondary antibodies in 3% Triton X-100/Dako REAL Antibody Diluent (S2022, Dako, Denmark) overnight at 4°C, washed with PBS 5 times, and then mounted with FluorSave Reagent (Millipore). Goat anti-mouse IgG Alexa Fluor 546 (Thermo Fisher, Waltham, MA, USA) and goat anti-rabbit IgG Alexa Fluor 647 (Thermo Fisher, Waltham, MA, USA) were used for pre- and post-synaptic marker visualization, respectively. Images were acquired on an inverted confocal microscope Leica-TCS SP8, with oil-immersion 63x objective magnification lens. Resolution of the image was 1024 pixels by 1024 pixels, and 5 sequential z-stacks with 0.3-μm intervals. The z-stack image was acquired by averaging 4 images on each z-plane with frame sequential method. For postnatal samples of B6J and *rd1* mice, the slices containing optic disc were used, and the area 500 μm away from the optic disc was imaged. We fixed the imaging area because retinal development proceeds from the central area to the periphery, and the timing of synaptogenesis might differ depending on the location. For post-transplantation samples of *rd1*, two randomly selected areas from slices containing transplanted graft were imaged.

For IHC of host-graft synapse evaluation, mouse anti-CACNA1s antibody (MAB427, Millipore, CA, USA) was used as the first antibody of the post-synaptic marker. Antigen retrieval was omitted in these samples in order to image GFP and tdTomato from the host bipolar cells and graft synaptic terminal CtBP2, respectively.

### Image Processing

Fiji (version 2.0.0-rc-65), an open source distribution of ImageJ (version 1.51s, NIH, USA) was used for image processing. IHC images were imported to Fiji, and 5 consecutive z-stack images from the upper edge of the sample were z-projected by averaging, to improve image quality and reduce noise. Protocols for DAPI, pre-synaptic and post-synaptic staining were optimized respectively as described below. More details including parameters of each functions are described in depth in Figures S1–S3.

#### [DAPI] (Figure S1)

For processing of the DAPI channel, the “Subtract background” function was used to reduce the background signal, and a bandpass filter was applied for reducing small particle noises. To select the area with signal, “Robust Automatic Threshold Selection” was applied followed by the “Dilate” function to slightly enlarge the selection. Next, “Adjustable watershed” was applied to the image to separate nuclei that have been merged together. Then, “Analyze particle” function was used to select the threshold area and generate regions of interest (ROIs), which were later used on the original image to extract graphical information from the unaltered image.

#### [Pre-synaptic Marker] (Figure S2)

First, the “Subtract background” function was used to reduce the background signal, then a bandpass filter was applied to the images for reducing small particle noises. The image was then smoothed by applying the “Smoothing” function, to make the signal within each region more homogeneous. Next, images were roughly segmented using the “Find Maxima” function with the “Segmented Particle” option. This separates the entire image into smaller segments based on local maxima, allowing us to extract all the regions regardless of signal intensity. Then, we performed a second “Find Maxima” function on each of the segments, but this time we used the “Maxima Within Tolerance” option for thresholding. The threshold area was then selected by the “Analyze Particle” function for later use as ROI. This sequential approach allowed us to have an adaptive threshold value based on the background intensity around each ROI.

#### [Post-synaptic Marker] (Figure S3)

A bandpass filter was applied to the images for reducing small particle noises. The post-synaptic marker mGluR6 has a punctate expression pattern in the ribbon synapse, and therefore we used the “Maximum filter” function to enhance the punctate signal. Then, images were processed the same way they were for the pre-synaptic marker. Briefly, images were segmented by the “Find Maxima” function with the “Segmented Particle” option, and adaptively thresholded in each segment, and ROIs were generated by the “Analyze Particle” function.

All generated ROIs were overlaid on the original z-projected image of each channel to extract 34 graphical parameters from each ROI of the unaltered image. Acquired ROI parameters were exported as a csv file for later use in the Naïve Bayes classifier.

All the processes described above were built into an ImageJ macro, so that images can be processed automatically, and multiple images can be processed in batch.

### Training Data of Synapse and Noise

The adult retina is organized into distinct layers, with photoreceptor/bipolar synapses located in the outer plexiform layer (OPL), an area that is clearly delineated by photoreceptor cell and bipolar cell nuclei. We prepared images from postnatal day (P) 28 B6J mouse retina containing only the OPL or excluding the OPL as training data for *Ideal Synapse* and *Ideal Noise*. Three to four replicate sections from three different mice were immune-stained and used as training data. The OPL area was manually cropped assuming that signals from this area originate mostly from synapses. The complement of OPL was used as noise teacher data, on the assumption that there are almost no photoreceptor/bipolar synapses outside the OPL. Note that although we call it noise, we do not necessarily mean or assume that these are non-specific staining or artifacts. In fact, both pre- and post-synaptic markers are known to be present outside the OPL. For example, RIBEYE is present in the inner plexiform layer (IPL) on the axonal terminal of bipolar cells as well as in the OPL (tom Dieck and Brandstätter, 2006); however, as its morphology and molecular component differs from that of the photoreceptor ribbon synapse (Heidelberger et al., 2005), its staining pattern also differs. Thus, although both IPL and OPL synapses are visualized by RIBEYE immunostaining, a careful examination of their signal can distinguish them. Furthermore, the ribbon synapse of IPL and OPL can be distinguished by its post-synaptic marker, because the retinal ganglion cell does not express mGluR6 in IPL. We therefore called staining patterns that resemble the adult photoreceptor ribbon synapse *Ideal Synapse*, and any other signals *Ideal Noise*, regardless of whether those noise signals represent a physiological or functional signal or not.

*Ideal Synapse* and *Ideal Noise* data were segmented and thresholded, and graphical information of ROIs was extracted as described in Figure 1 and Figures S1–S3. We categorized the extracted parameters into three categories: *Signal, Morphology*, and *Geometry*. *Signal* parameters include a series of measurements that represent the characteristics of staining signal including: *mean, median, mode, minimum, maximum, standard deviation, skewness*, and *kurtosis*. In addition to the raw value of these parameters, all the *Signal* parameters, except for *skewness* and *kurtosis*, were divided by a global background intensity to compensate for variance of IHC background intensity. The global background intensity was calculated from the pixel intensity of the entire image. The background was summarized as the peak value of the signal intensity distribution estimated using Kernel Density Estimation (KDE). Signals below the intensity of 8 were ignored in the peak estimation assuming they represent areas where there was no tissue. *Morphology* parameters include *perimeter, width, height, shape, major, minor, angle, AR, round, circularity, solidity, ferret, minferet, angle*, and *feret angle*. Perimeter represents the length of outer edge of the ROI. *Width* and *height* represent horizontal and vertical length of bounding box that can fit ROI. *Shape* parameter is our original parameter which is represented by $shape=\;{\sqrt{\left\{XM-\left(BX-\frac{width}{2}\right)\right\}}}^{2}+{\sqrt{\left\{YM-\left(BY-\frac{height}{2}\right)\right\}}}^{2}$ where XM and YM represents coordinates of brightness-weighted center of mass, and BX and BY represents coordinates of upper-left corner of rectangle. *Major* and *Minor* are the longer and shorter axis when the ROI was fitted with ellipsoid. *Angle* is the angle between the longer axis of the ROI and the horizontal line. *AR* is the aspect ratio of width and height of bounding box. *Feret* angle represents the angle of *ferret. minferet* represents the longest and shortest diameter of the ROI. Geometrical parameters include the distance between pre- and post-synaptic markers, the angle of the pair, the area of each synaptic marker, and the raw integrated density which represents area and intensity simultaneously.

Using the training data, we generated the Probability Density Functions (PDFs) of *Ideal Synapse* and *Ideal Noise* for each of the parameters (Figure S5). PDFs were estimated for each parameter from their histograms, either by Kernel Density Estimation (KDE), or by Bounded Density Estimation (BDE) for parameters that had a clear boundary.

### Naïve Bayes Classifier

The Naïve Bayes classifier is a simple but robust classifier algorithm, which employs the Bayes theorem to estimate the posterior probability using the prior probability and likelihood based on training data.

Naïve Bayes classifier used in QUANTOS can be represented as follows:

$$\begin{array}{l} p(C_{i}|x_{1}\ldots x_{n})=\frac{p\left(C_{i}\right)p\left(x_{1}\ldots x_{n}|C_{i}\right)}{p\left(x\right)}\;\\ \;(\text{where }i=synapse\;or\;noise) \end{array}$$

*p*(*C*_*i*_|*x*_1_ … *x*_*n*_) represents posterior probability of being either synapse or noise, given *n* different parameters *(x)*. *p*(*C*_*i*_) represents prior probability of synapse or noise, *p*(*x*_1_ … *x*_*n*_|*C*_*i*_) represents likelihood of synapse or noise under condition of parameters *x*, and *p*(*x*) represents evidence.

#### [Prior Probability]

Prior probability was estimated from marker density, on the assumption that the presence of more markers decreases the probability of correctly identifying synapses. We generated two sets of points randomly within a square area at different densities to simulate the behavior of non-specific pre- and post-synaptic markers. This simulation shows that the number of randomly generated pairs within a certain distance is proportional to the density of the markers (Figure S4A). This can clearly be visualized in Figure S4A, where the number of random pairs increases with marker density. The slope of the regression line, which we termed “random factor”, is in a quadratic relationship with the maximum distance of pairs (Figure S4C). Thus, the number of pairs formed by chance (i.e., random pairs) can be estimated from the marker density and the random factor with the following equation:

*random pairs* = (*random factor*) × (*density of pre*) × (*density of post*)

#### (Figure S4D)

A 5 μm × 5 μm square area around each of the center coordinates markers was used for estimating pre- and post-synaptic marker density.

Having estimated the number of random pairs, the prior synapse probability is estimated as:

$$\begin{array}{l} prior\;synapse\;probability=0.5\;\left(\text{if }random\;pairs<2\right)\\ \;prior\;synapse\;probability=\frac{1}{random\;pairs}\\ \;\left(\text{if }random\;pairs\geq 2\right) \end{array}$$

The prior probability for noise is simply

$$\begin{array}{l} prior\;noise\;probability=\;1-\;prior\;synapse\;probability \end{array}$$

Thus, the priors for synapse and noise are equal if the number of markers is low, but the synapse prior decreases as the number or markers increases.

#### [Likelihood]

The likelihood is given by the Probability Density Function (PDF) of the *Ideal Synapse* and the *Ideal Noise* data. Pre- and post-synaptic markers are evaluated separately, and the total likelihood of synapse candidate pair is estimated by multiplying their individual likelihoods. Pre- and post-synaptic markers whose centroid coordinates were within 1.2 μm were assigned as possible synapse candidates. The distance threshold was decided based on the *Ideal Synapse* data set, where synapses were most often observed around 0.51 μm with a standard deviation of 0.17 μm. After selecting the synapse candidates, the Naïve Bayes classifier was used to estimate the likeliness of each synapse candidate being synapse or noise. Evidence is likewise calculated from the joint synapse and noise likelihoods, by the following:

$$\begin{array}{l} \left(prior\;synapse\;probability\;\times \;likelihood\;of\;synapse\right)\\ +\;\left(prior\;noise\;probability\;\times \;likelihood\;of\;noise\right) \end{array}$$

### Analysis of *rd1* Mice After miPSC-Retina Transplantation

Host cells and transplanted cells were distinguished by identifying the remaining retinal ganglion cell layer (GCL) and INL of the host by morphology. The area encompassing the transplanted cells was manually traced to evaluate synapse formation in transplanted cells.

Unlike postnatal development of the B6J mouse, the number of transplanted photoreceptors is not homogenous among samples. Therefore, the numbers of photoreceptors were quantified in transplanted samples to estimate the number of synapses per photoreceptor. Transplanted photoreceptors can be identified by their nuclei shape, characteristic of photoreceptor cells, and by the formation of dome-like structures called rosettes. For quantification of photoreceptors, the area of rosette forming cells was manually selected in each image, then the number of DAPI ROIs contained in that area was analyzed using the protocol for DAPI analysis described above in the “Image Processing” section.

### Average Synapse

All detected synapses were individually cropped to a 4.34 by 4.34 μm square, with the center coordinates of the synapse in the center of the square. Ribbon synapses can be formed at various angles (Figure 2D) and thus images were rotated to align the center coordinates of pre- and post-synaptic markers, using the angle of the line connecting the pre- and post-synaptic markers. Then all synapses from each postnatal day and each rearing condition were averaged. For analysis of average synapse data, we used “Radial Profile” of ImageJ Fiji, which exports the intensity along the distance from the center coordinates. The center coordinates of averaged images were estimated using the “Find Maxima” function for both pre- and post-synaptic markers.

### Micro-Electroretinography (mERG)

The mERG was conducted using the multi-electrode array (MEA) recording system (USB-MEA60-Up-System, MultiChannel Systems, Germany) with the standard 8x8 probe (60MEA200/30iR-Ti-gr) as previously described (Iraha et al., 2018). In order to distinguish the effects of prolonged dark adaptation from dark rearing, mice reared under LD and LL conditions were dark adapted for 24 h prior to the recording, as long dark adaptation (6 to 24 h) can significantly reduce the b-wave amplitude (Li et al., 2016). P14 B6J mice were deeply anesthetized with sevoflurane inhalation, followed immediately by decerebration and harvest of retinas. After removal of the vitreous body, retinas were mounted on electrodes with the ganglion cell side down and constantly supplied with warmed (35 ± 0.5°C), carbonated (95% O_2_ and 5% CO_2_) Ames' medium (A1420, Sigma-Aldrich) perfused at 3–3.5 mL/min. Opsinamide (10 μM; AA92593, Sigma-Aldrich) was added in the perfusion medium to suppress the melanopsin-driven RGC light responses during recording. Retinas were allowed to recover in the MEA chamber for at least 20 min before recording. Field potentials to full-field white light stimuli were recorded at 20 kHz. The 10 ms full-field light stimulus was generated using a white LED source with an irradiance of 10.56 log photons/cm^2^/s at the focal plane of the electrodes, which approximated the low mesopic range of mature wildtype mouse vision. All of the above procedures were conducted under dim LED light with a peak wavelength at 690 nm.

mERG traces were processed and analyzed in R (R. C. Team - Austria: R Foundation for Statistical Computing Google, 2017). A band-pass Butterworth filter (1–50 Hz) was applied to traces to remove low frequency fluctuations and high frequency jitter. Local minima within 55 ms from light stimulation were flagged as a-wave, and local maxima within 150 ms from light stimulation were flagged as b-wave. The a-wave amplitude was calculated from the baseline, and the b-wave amplitude was calculated from the a-wave, or from the baseline when the a-wave was not detected. Replicates from three repeated stimulations were averaged.

### Statistical Analysis

We used full Bayesian statistical inference with MCMC sampling for statistical modeling. Bayesian inference was implemented in Rstan (Stan Development Team, 2017). We estimated population effects, such as the effect of light, individual differences, as well as experimental variation.

Posterior distribution of parameters of interest, which show the most likely values given the data, are shown with 89% confidence intervals. When the difference between conditions is of interest, we show the difference of posterior distributions expressly, as posterior distributions may be correlated or anticorrelated. When the 89% confidence interval of difference of posterior distributions does not cross over zero, estimated parameters are considered different.

#### [Developmental B6J Mouse Analysis]

We parameterized developmental synaptogenesis with a modified (Gompertz) growth curve (Zwietering et al., 1990), which is defined by three parameters describing the onset of synaptogenesis (λ), the maximum rate of synaptogenesis (μ) and the maximum capacity of synapse (A). Acquired data was analyzed with the following multilevel model:

$$\begin{array}{l} y_{i}\sim Poisson\left(\Lambda_{mouse}\right)\\ \Lambda_{mouse\;}\sim Gamma\left(\Lambda_{condition,\;day\;},\;\beta \right)\\ \Lambda_{condition,\;day}\sim Gompertz\left(A_{condition,\;}\mu_{condition},\;\lambda_{condition},\;day\right) \end{array}$$

Each observation *y*_*i*_ is a count data, so we assumed a Poisson distribution with a mean Λ_*mouse*_, representing the average number of synapses for the sampled mouse. We assumed a Gamma distribution for mice sampled on the same postnatal days, as the average number of synapses should be a positive number. The expected number of synapses is given by the Gompertz growth curve given the rearing condition (DD or LD) and postnatal day.

#### [mERG Analysis]

B-wave amplitude was analyzed with a multilevel generalized linear model using the Gamma distribution as likelihood, as b-wave amplitude is always a positive value and data was spread with a long tail toward larger values.

$$A_{n\;}\sim \;Gamma\left(\mu_{n}\;,\sigma_{n}\right)\;n=1,\ldots ,\;N$$

where *A*_*n*_is the b-wave amplitude of the n-th observation, μ is the mean, and σ represents the standard deviation. We parameterized with the mean and standard deviation rather than the shape and rate parameters, in order to place informative priors and to make the interpretation more intuitive. The different rearing conditions and the animals from which samples were obtained were used as predictors with the exponential link function.

$$\begin{array}{lll} \mu & = & \exp\left(a_{0}+a_{condition}+a_{mouse}\right)\\ \sigma & = & \exp\left(b_{0}+b_{condition}+b_{mouse}\right) \end{array}$$

#### [Synapse Number After Transplantation]

The number of synapses per photoreceptor cell on post-transplantation samples was compared using the Student-t distribution for robust Bayesian estimation assuming equal variance between conditions. Thus each observation (number of synapses per photoreceptor cells) *y*_*i*_ is distributed as

$$\begin{array}{l} y_{i}\sim Student\text{\_}t\left(\upsilon ,\;\mu_{i},\;\sigma \right) \end{array}$$

where υ is the normality parameter, μ is the mean, and σ is the standard deviation. The different rearing conditions and the animals from which samples were obtained were used as predictors.

$$\begin{array}{l} \mu_{i}=a_{o}+a_{condition}+a_{mouse} \end{array}$$

[ROC analysis]

*Ground Truth* for P14 and P28 samples were generated by careful classification by an expert observer. Coordinates of QUANTOS result and *Ground Truth* were considered as “matched” when they were within 1 μm. ROC curve analysis was conducted using the “pROC” package in R (Robin et al., 2011). The ROC curve was drawn in reference to the *Ground Truth* to evaluate the sensitivity and specificity of QUANTOS.

### Macros and Scripts

ImageJ Fiji macro and R scripts used in this manuscript are available at: https://github.com/matsutakehoyo/QUANTOS

## Author Contributions

RA, TM, MT, and MM conceptualized the study. RA and TM developed QUANTOS and conducted the statistical analysis. RA conducted immunohistochemistry. RA, MM performed subretinal transplantation. H-YT conducted electrophysiology. TH maintained and differentiated iPSC retina. JS cared for animals and assisted on transplantation. MT and SY oversaw the study. RA, TM, H-YT, and MM wrote the manuscript with input from all authors.

### Conflict of Interest Statement

The authors declare that the research was conducted in the absence of any commercial or financial relationships that could be construed as a potential conflict of interest.

## Supplementary Material

Figure S1 describe image processing protocols for DAPI, RIBEYE, and mGluR6 channel, respectively. Immunostaining samples were imaged with a confocal microscope and 5 sequential z-stack images were projected on the z axis, and processed as described in the Figure. After generating ROIs, the following graphical parameters were exported using the “Measure” function of ImageJ Fiji: *Area, Mean, StdDev, Mode, Min, Max, X, Y, XM, YM, Perim, BX, BY, Width, Height, Major, Minor, Angle, Circ, Feret, IntDen, Median, Skew, Kurt, RawIntDen, FeretX, FeretY, FeretAngle, MinFeret, AR, Round, Solidity*.

Figure S2 Same as Figure S1.

Figure S3 Same as Figure S1.

Figure S4 Estimation of marker density. **(A)** Relationship between pre-synaptic marker density, post-synaptic marker density and the number of pairs when the maximum distance of pairs is 1 μm. **(B)** Modified Figure of **(A)** with the y axis divided by post-synaptic density. **(C,B)** With different maximum distance of pairs. **(D)** As distance of pairs increase, the chance of random pairs increase. Therefore, we used density of markers as prior probability to lower the prior probability when marker densities are high as described in [Prior probability] of Methods section.

Figure S5 Probability density functions of parameters. PDFs were generated by *Ideal Synapse* and *Ideal Noise* training data. Likelihoods based on each PDF were used upon estimating the posterior probability of synapse for each synapse candidate. Angle and distance histograms of all the detected synapses are also shown here in the right upper panel. Insets show IHC images of detected synapses at various angles. Left upper panel show PDFs of *Geometry* parameters, middle panel show PDFs of *Morphology* parameters, and lower panel show PDFs of *Signal* parameters. PDF, probability density function.

Figure S6 Example of QUANTOS maturity evaluation Sample IHC image of B6J P14 **(A)** and P21 **(C)** mouse. On P14, both mature and immature synapses can be found. Scale bar = 10 μm Yellow box area of **(A)** and **(C)** were magnified and QUANTOS results are overlaid in **(B)** and **(D)** respectively. Upper panels show all synapses detected by QUANTOS with yellow dots, including immature and mature synapses. Middle panels show mature synapses detected by QUANTOS with magenta circles. Lower panels show both all synapses and mature synapses detected by QUANTOS. Scale bar = 5 μm.
