# Supplementary figures and images for: Quantitative and Qualitative Evaluation of Photoreceptor Synapses in Developing, Degenerating and Regenerating Retinas
Source: Front Cell Neurosci. 2019 Feb 11;13:16. doi: 10.3389/fncel.2019.00016 (PMC6378395; doi:10.3389/fncel.2019.00016)

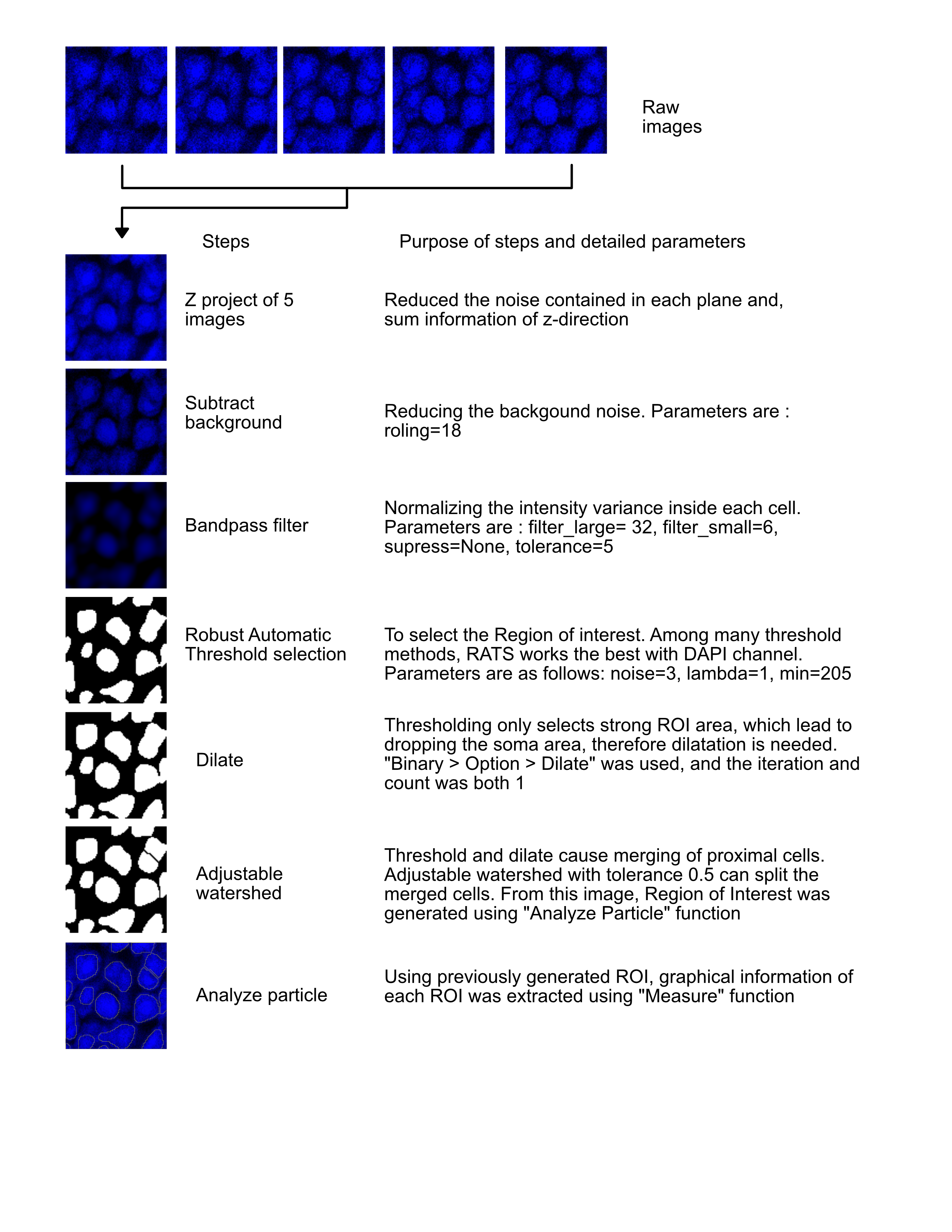

Supplement: Figure S1 — describe image processing protocols for DAPI, RIBEYE, and mGluR6 channel, respectively. Immunostaining samples were imaged with a confocal microscope and 5 sequential z-stack images were projected on the z axis, and processed as described in the Figure. After generating ROIs, the following graphical parameters were exported using the “Measure” function of ImageJ Fiji: Area, Mean, StdDev, Mode, Min, Max, X, Y, XM, YM, Perim, BX, BY, Width, Height, Major, Minor, Angle, Circ, Feret, IntDen, Median, Skew, Kurt, RawIntDen, FeretX, FeretY, FeretAngle, MinFeret, AR, Round, Solidity. [file Image_1.TIFF]

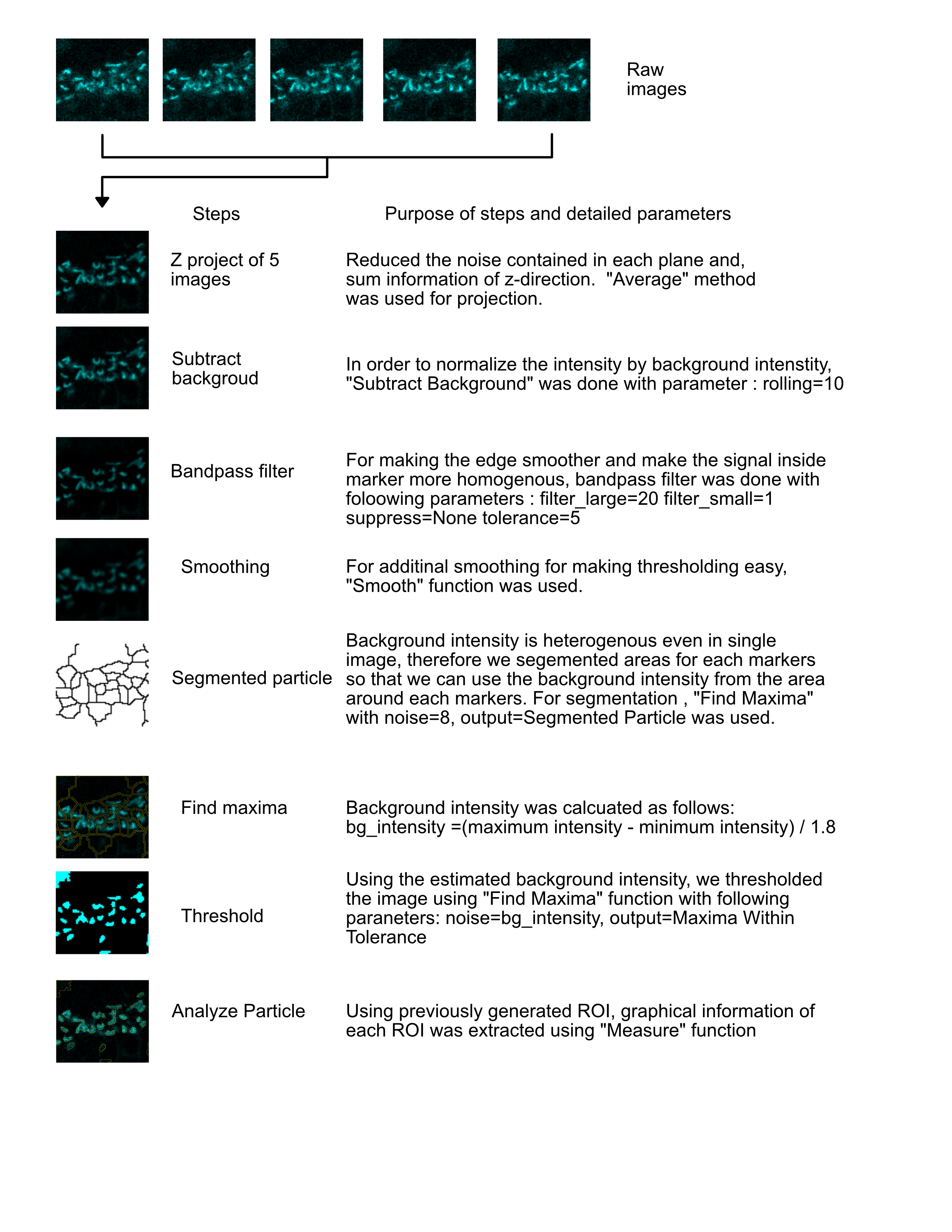

Supplement: Figure S2 — Same as Figure S1. [file Image_2.TIFF]

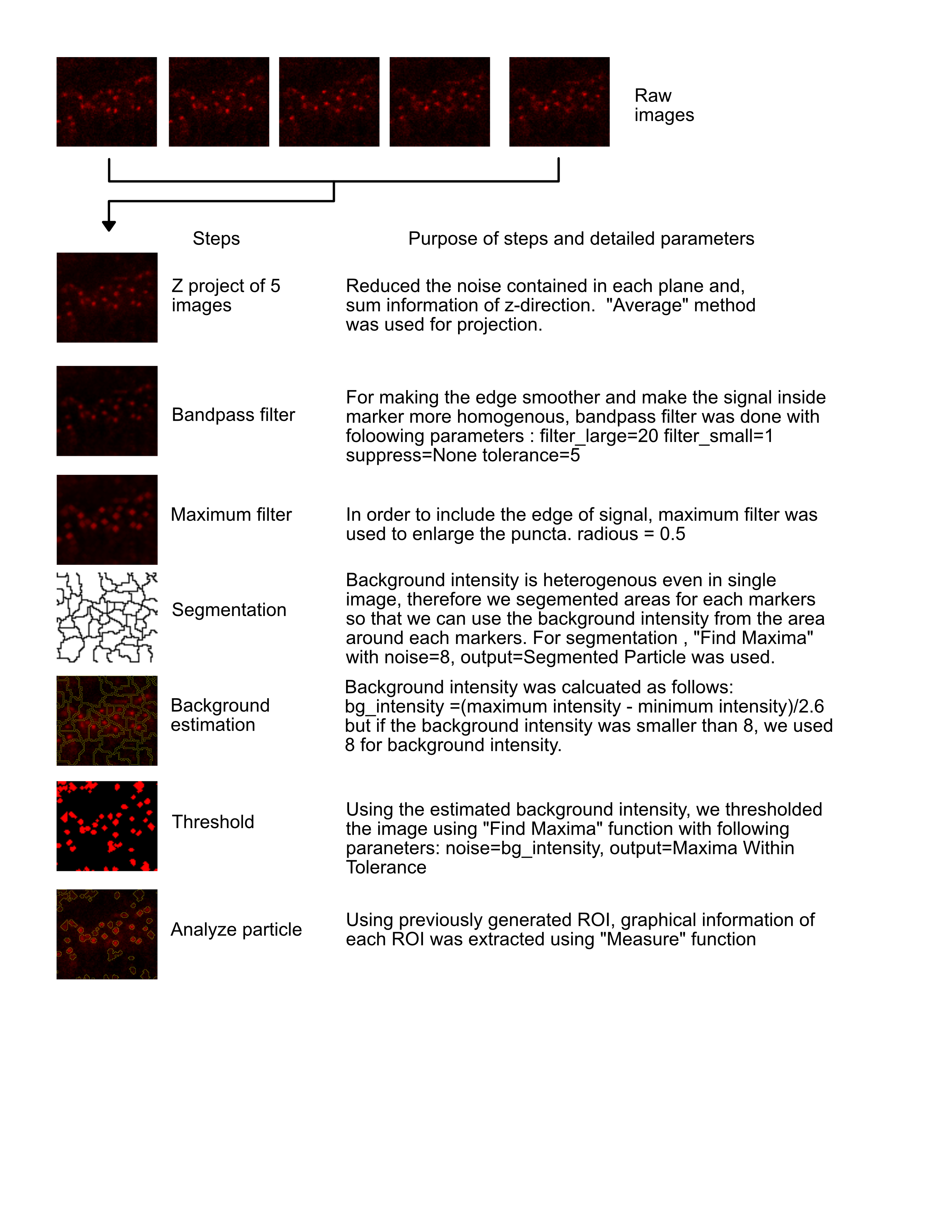

Supplement: Figure S3 — Same as Figure S1. [file Image_3.tiff]

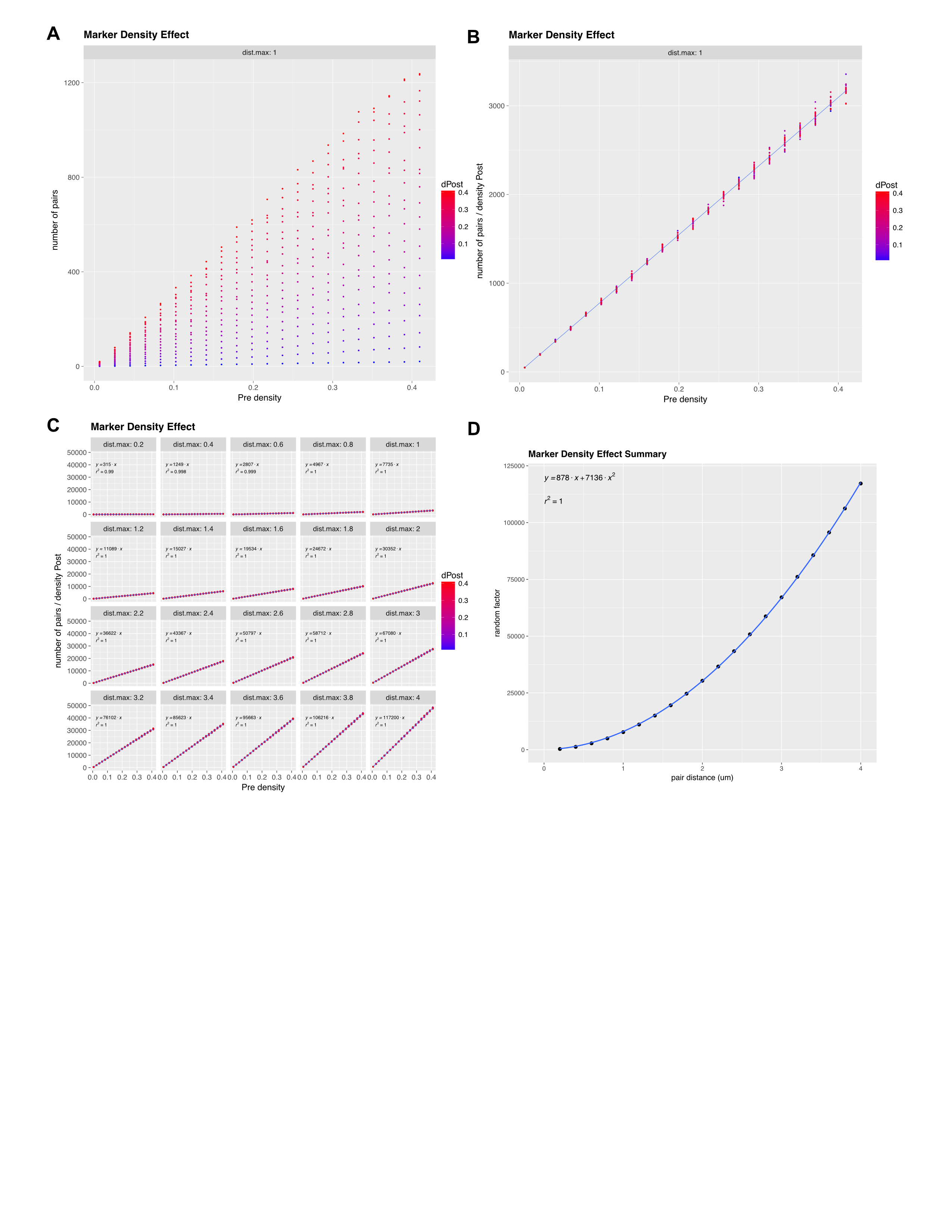

Supplement: Figure S4 — Estimation of marker density. (A) Relationship between pre-synaptic marker density, post-synaptic marker density and the number of pairs when the maximum distance of pairs is 1 μm. (B) Modified Figure of (A) with the y axis divided by post-synaptic density. (C,B) With different maximum distance of pairs. (D) As distance of pairs increase, the chance of random pairs increase. Therefore, we used density of markers as prior probability to lower the prior probability when marker densities are high as described in [Prior probability] of Methods section. [file Image_4.tiff]

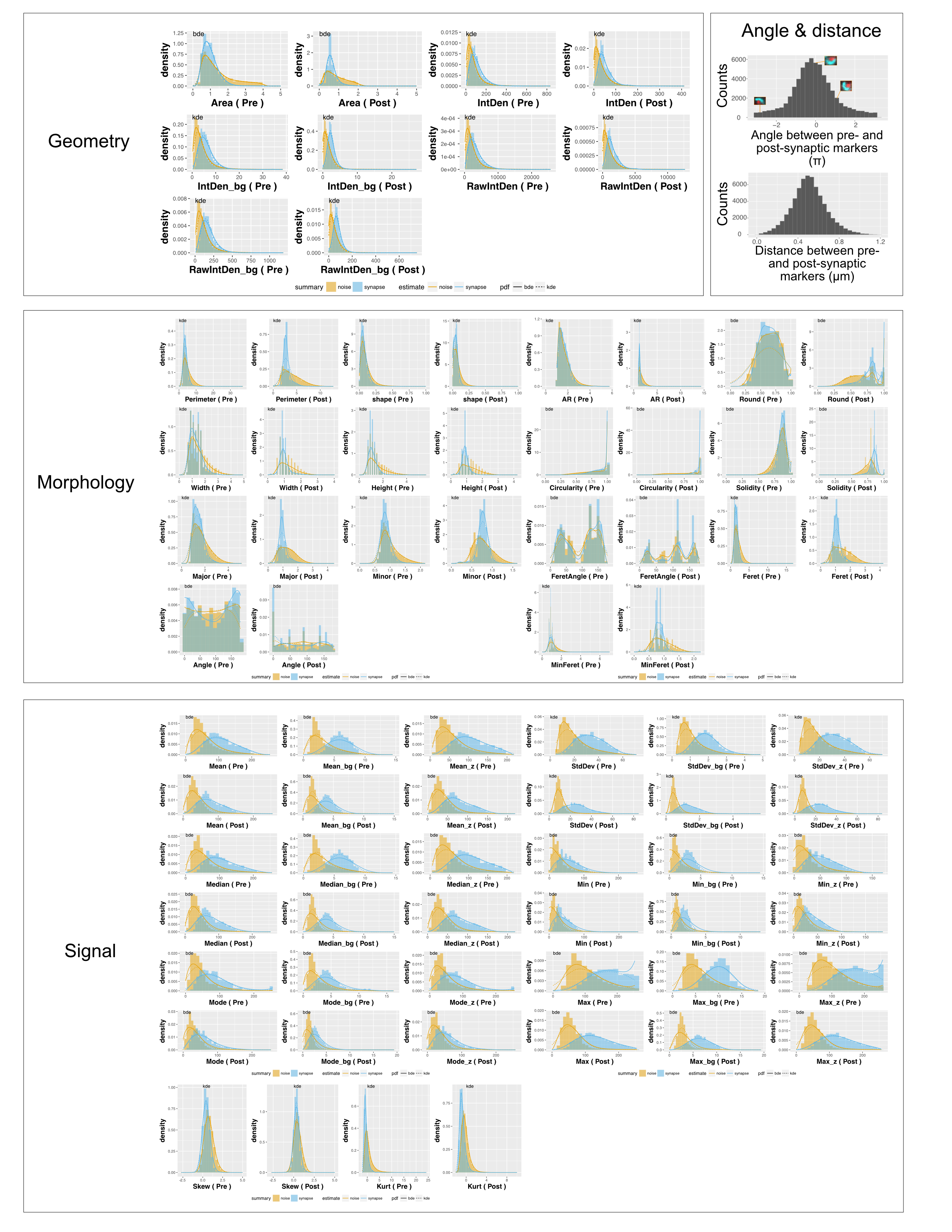

Supplement: Figure S5 — Probability density functions of parameters. PDFs were generated by Ideal Synapse and Ideal Noise training data. Likelihoods based on each PDF were used upon estimating the posterior probability of synapse for each synapse candidate. Angle and distance histograms of all the detected synapses are also shown here in the right upper panel. Insets show IHC images of detected synapses at various angles. Left upper panel show PDFs of Geometry parameters, middle panel show PDFs of Morphology parameters, and lower panel show PDFs of Signal parameters. PDF, probability density function. [file Image_5.TIFF]

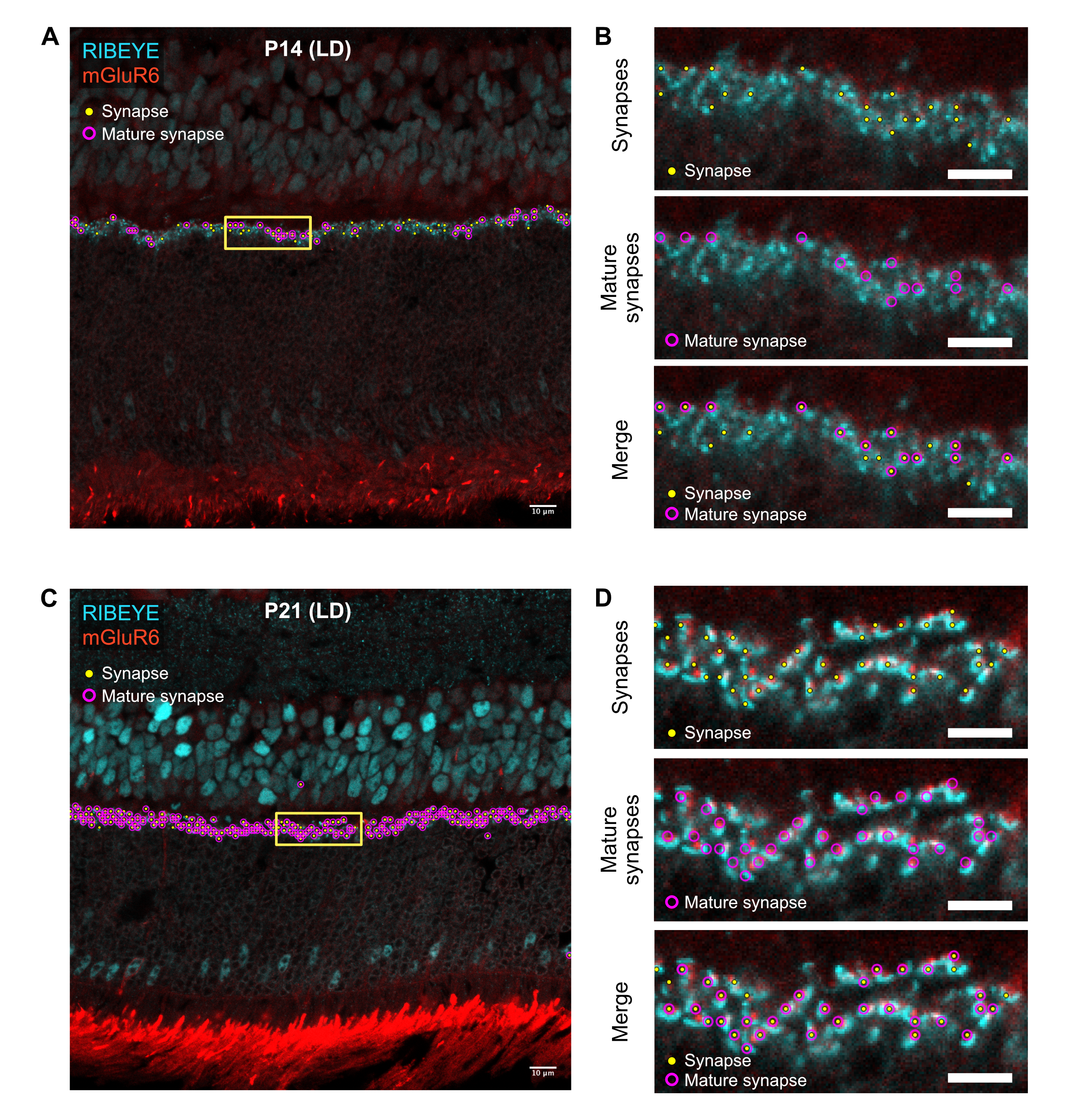

Supplement: Figure S6 — Example of QUANTOS maturity evaluation Sample IHC image of B6J P14 (A) and P21 (C) mouse. On P14, both mature and immature synapses can be found. Scale bar = 10 μm Yellow box area of (A) and (C) were magnified and QUANTOS results are overlaid in (B) and (D) respectively. Upper panels show all synapses detected by QUANTOS with yellow dots, including immature and mature synapses. Middle panels show mature synapses detected by QUANTOS with magenta circles. Lower panels show both all synapses and mature synapses detected by QUANTOS. Scale bar = 5 μm. [file Image_6.tiff]
